# Effects of landscape pattern on land surface temperature in Nanchang, China

**DOI:** 10.1038/s41598-024-54046-4

**Published:** 2024-02-15

**Authors:** Pinyi Liu, Chunqing Liu, Qingjie Li

**Affiliations:** 1 School of Landscape and Art, Jiangxi Agricultural University, Nanchang, 330045 China; 2 Jiangxi Rural Culture Development Research Center, Nanchang, 330045 China

**Keywords:** Ecology, Environmental sciences, Environmental social sciences

## Abstract

The composition and configuration of landscapes are critical important to design effective approaches to mitigate urban thermal environment in the urbanization process. In this research, land use maps and land surface temperature (LST) retrieval were derived in Nanchang city of central China based on product datasets and the thermal infrared band of Landsat. The results showed that the thermal environment of Nanchang had become worse over the past two decades, that is, the proportion of area of the extremely low temperature zone (ELTZ) decreased from 4.39 to 0.77% from 2001 to 2020, and that of medium temperature zone (MTZ) reduced by 20%, whereas those of the high temperature zone (HTZ) and the extremely high temperature zone (EHTZ) increased sharply after 2001, and by 2020, the area ratio increased by 11% and 7.16%, respectively. The agricultural land (AL) area decreased from 68.44 to 49.69%, was gradually replaced by construction land (CL). The CL occupied the largest proportion in EHTZ, HTZ and slight high temperature zone (SHTZ); water landscape (WL) and green land (GL) occupied the largest proportion in ELTZ, low temperature zone (LTZ); and AL occupied the largest proportion in SHTZ, MTZ, and slight low temperature zone (SLTZ). Landscape configuration also obviously impacted on LST. The model fitting was well (R = 0.87) between land use area and LST by multiple regression analysis. The significant correlation between LST and six landscape pattern indices of CL (*p* < 0.01) indicated that the larger percent (PLANT, R = 0.78) and the more concentrate (LPI, R = 0.73) of CL implied the higher LST, while the more fragment (NP, R = − 0.45), dispersed and complex shape (R = − 0.35) were benefit to relieve LST. Contrastively, the larger percent and the more concentrated and complex shape distribution of AL, GL and WL, the lower LST (*p* < 0.01). In addition, LST had closely correlation with landscape level indices such as aggregation degree (AI, R = 0.44) and diversity (SHDI, R = − 0.60) (*p* < 0.01).

## Introduction

According to the goals set out in the 14th Five-Year Plan (2021–2025) for national economic and social development of China, 65% of the population lives in urban areas. In the next two to three decades, China's urbanization population will gradually rise to more than 80%. The continuous improvement of urbanization is also ongoing in Asia and Sub-Saharan Africa^1,2^. Urban expansion leads to the variation of land use (land cover) types and results in a differential heating process^3^. For instance, the increasing expansion and intensification of natural surface transferred to impervious surface are considered to enhance urban heat island (UHI) effect, because impervious surface can capture heat and lessening evaporative cooling in contrast to naturally surface^4,5^. With the advance of global warming, extreme hot weather occurs frequently, which further exacerbates the heat environment of urban^6,7^. UHI can causes negative environmental and health impacts^8^, such as urban polluted air^9,10^, more energy and water consumption^11,12^, and extreme heat-related morbidity and mortality^13^.

The land cover changes (urban growth), such as the conversion of natural surfaces to impervious surfaces, often result in land surface temperature (LST) rise, referred as the main cause of the UHI effect^14^ Data of LST can be effectively obtained based on remote sensing in large scale, real time, and continuous manner in the recent decades^15^. The development of landscape ecology has facilitated to research urban heat environment. Landscape pattern metrics of land cover can provide a comprehensive description of heat island pattern from landscape composition and spatial arrangements^16^. Landscape composition is described as the proportion of each land cover type, including green space, water body, construction land and barren land^7,17,18^, which has been applied to research its relationship with LST based on correlation analysis^3,19^. In addition, landscapes configuration (such as size, number, shape of patch) also obviously affects the magnitude of LST^15,16^. Some studies have indicated that landscape configurations are more important factor influencing the cooling effect of water and green space than landscape composition^20,21^. However, these studies have commonly focused on a discrete area of landscape and the effects of the area's size and shape, rather than the spatial arrangement of different landscape types^17^. Other spatial landscape metrics such as distance, interspersion, patch density, aggregation, contagion, diversity are rarely used to describe the variation of LST from the overall characteristics of different land cover^22^. Hence, the role of spatial configuration of different land cover in affecting LST should be further quantified^23^, and it would be necessary and helpful for urban planning.

This research focused on the dynamics of LST and land use types in the urbanization process during 2001 and 2020 based on Landsat and various geospatial approaches. The purpose were: (1) to investigate the role of landscape composition and configuration in indicating LST; (2) to quantified the coupling relationships between LST and landscape pattern of different land cover. The results can provide a reference for better managing the land use changes and landscape planning within cities in order to mitigate the thermal environment.

## Materials and methods

### Study sites

**Figure 1:**
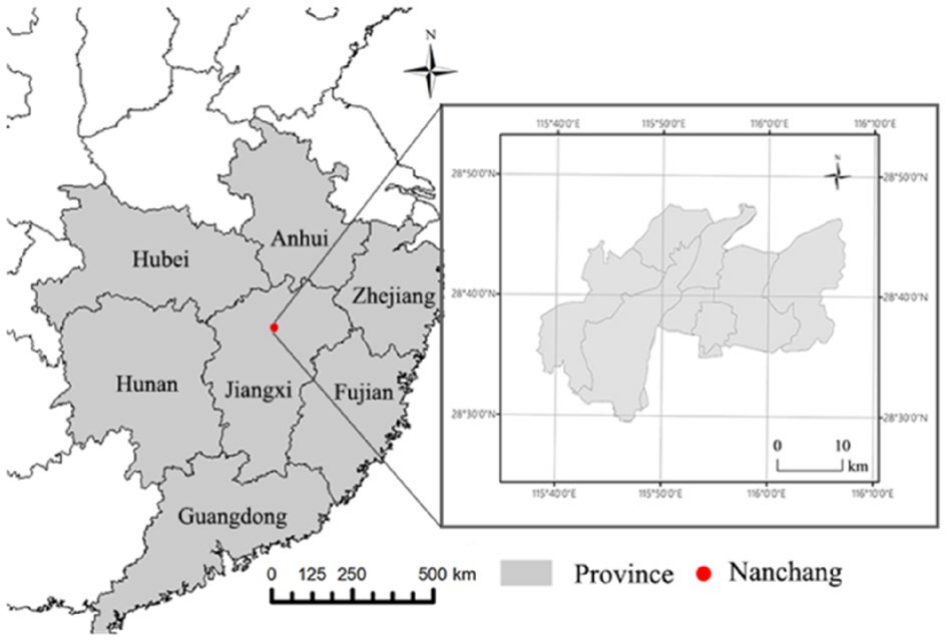
Location map of urban Nanchang and adjacent provinces in China.

Nanchang is an inland urban (Fig. 1), the capital of Jiangxi with population of 6 millions (as of 2021), is considered as the “furnace” city in central China with air temperature exceeds 30–40 °C in summer. The mean annual temperature and precipitation in Nanchang is approximately 23 °C and 1600 mm. The landscapes of the urban are a mosaic of built-up (construction) land, cropland, grassland, and water body including parts of the lakes, rivers and ponds. This study was conducted using data from 2001 to 2020, a period of the typical developing stage of urbanization in China^15,22^.

### Data processing

Thermal remote sensing (RS) has been used as a powerful tool in the exploration of LST for its broad spatial coverage, frequent revisit cycle and multiple data source^23–25^. The RS images (https://www.gscloud.cn/) applied in this research were selected from 13/5/2001, 16/5/2008, 1/5/2014, 15/4/2020 and 6/9/2020 under the stability of weather conditions (gentle breeze and sunny) when the satellite had to pass the same location^26,27^.

### LST retrieval

The radiative transfer equation (RTE) method is applied to invert the land surface temperature (LST), can reach an accuracy of 0.6°C^28^. The software ENVI5.3 is applied for radiometric calibration and atmospheric correction of the raw satellite image^29^, and AicGIS10.2 is used to crop the study area. The normalized vegetation index (NDVI) and vegetation coverage (FVC) are used to calculate the surface emissivity (ε_i_). The formula was as follows:

1 $$FVC = \frac{{{\text{NDVI}} - {\text{NDVI}}_{{{\text{soil}}}} }}{{{\text{NDVI}}_{{{\text{veg}}}} - {\text{NDVI}}_{{{\text{soil}}}} }}$$

2 $${\upvarepsilon }_{{\text{i}}} = 0.004{\text{FVC}} + 0.986$$

where NDVI_soil_ and NDVI_veg_ are the normalized vegetation index of the non-vegetated area and the normalized vegetation index of the total vegetated area, respectively. During atmospheric correction, the atmospheric radiation is simulated based on the principle of the RTE by the model in MODTRAN4.0^15^. Through this simulation, the downward and upward atmospheric radiance L_i_↓, L_i_↑ and atmospheric transmissivity τ_i_(θ) can be estimated. With ε_i_, the radiation intensity B(T_s_) can be obtained by Eq. (3). Consequently, the LST (T_s_) can be calculated by Eq. (4). More detailed process can be checked in the reference^15,30^.

3 $$B_{{\text{i}}} (T_{{\text{s}}} ) = [B_{i} (T_{i} ) - L_{i} \uparrow - \tau_{i} (\theta ) \cdot (1 - \varepsilon_{i} ) \cdot L_{i} \downarrow ]/\tau_{i} (\theta ) \cdot \varepsilon_{i}$$

4 $$T_{S} = \frac{{{\text{k}}_{2} }}{{\ln \left( {1 + \frac{{k_{1} }}{{B(T_{s} )}}} \right)}}$$

where L_i_ is the radiation intensity (W·m^−2^·sr^−1^·µm^−1^) of the wave band with index number i measured by the satellite sensor. K_1_ (unit mW·m^−2^·sr^−1^·µm^−1^) and K_2_ (unit K) are preset constants before the launch of the satellite (Table 1).

**Table 1:** The value of K_1_ and K_2_.

Landsat	K_1_	K_2_
Landsat 5 TM (Band6)	607.76	1260.56
Landsat 7 ETM + (Band6)	666.09	1282.71
Landsat 8 OLI (Band10)	774.89	1321.08
Landsat 8 OLI (Band11)	480.89	1201.14

### Classification of LST

After retrieval, LST are classified into seven types as Extremely High Temperature Zone (EHTZ), High Temperature Zone (HTZ), Slight High Temperature Zone (SHTZ), Medium Temperature Zone (MTZ), Slight Low Temperature Zone (SLTZ), Low Temperature Zone (LTZ), and Extremely Low Temperature Zone (ELTZ) with the method of standard deviation classification^15,31^. The calculation of the boundaries is shown in Eq. (5).

5 $${\text{D}} = {\text{X }} \pm {\text{ a }} \times {\text{ S}}$$

where D is the boundary of each type, a is times of 0.5 from 0 to 2.5, X is the average LST of the image, and the value S is the standard deviation. The seven types are listed in Table 2. The X and s for the four years were calculated separately.

**Table 2:** The classification of the land surface temperature.

Temperature grade	The temperature threshold
Extremely Low Temperature Zone (ELTZ)	D ≤ x – 2.5 s
Low Temperature Zone (LTZ)	X − 2.5 s < D ≤ x − 1.5 s
Slightly Low Temperature Zone (SLTZ)	X − 1.5 s < D ≤ x − 0.5 s
Medium Temperature Zone (MTZ)	X − 0.5 s < D ≤ x + 0.5 s
Slightly High Temperature Zone (SHTZ)	X + 0.5 s < D ≤ x + 1.5 s
High Temperature Zone (HTZ)	X + 1.5 s < D ≤ x + 2.5 s
Extremely High Temperature Zone (EHTZ)	D > x + 2.5 s

### Classification of land use

The land use maps were derived from the open-source datasets (http://www.resdc.cn). This dataset with a spatial resolution of 30 m were extracted from Landsat satellite imageries by supervised classification. High resolution images from Google Earth™ were used as the reference layers to assess the classification accuracy^23,32^. In this study, the land use at 30-m resolution in 2001, 2008, 2014 and 2020 was classified into five major landscape types: construction land (CL), green land (GL), water landscape (WL), agricultural land (AL), and barren land (BL).

### Landscape pattern index

**Figure 2:**
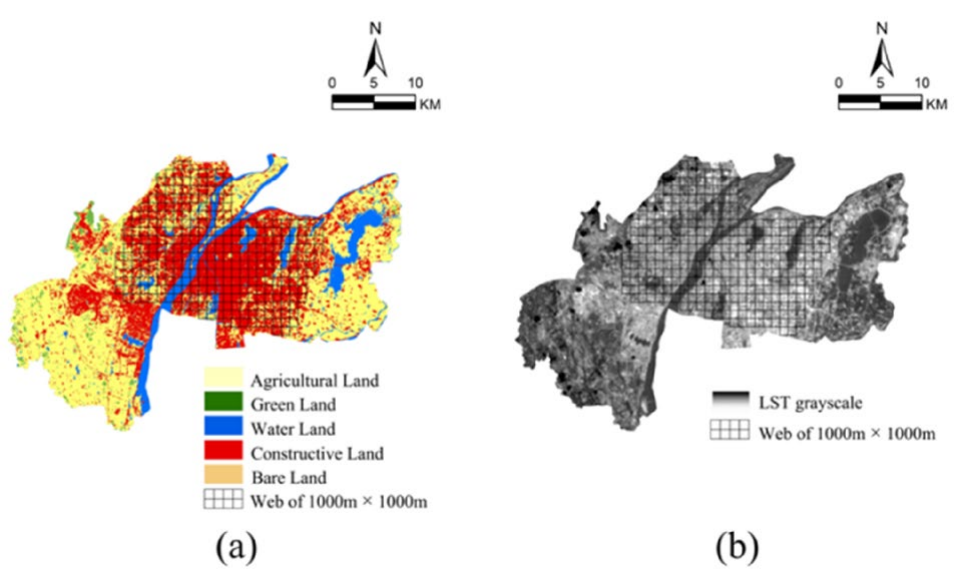
(**a**) Slice of land use type figure (**b**) Slice of LST grayscale figure.

The surface coverage data was calculated using 1000 m × 1000 m gridded fishnet on the ArcGIS 10.2 software platform for the surface coverage weighted center identification^33^. A total of 333 randomly selected polygon grids for study area were used to clip the study area's land cover map in 2020 (Fig. 2a,b). Eleven landscape pattern indices from the perspective of landscape ecology were selected to quantitatively describe the landscape pattern characteristics of land coverage in this study area. These indices are mainly from the two aspects of patch level and landscape level^34,35^. The patch level indices focus on the analysis of the number, morphology and structure of land use, including number of patch (NP), percent of landscape composition (PLAND), largest patch index (LPI), landscape shape index (LSI), mean closest distance (MNN), interspersion and juxosition index (IJI). The landscape level indices are used to describe the overall characteristics of land use status including patch density (PD), aggregation index (AI), contagion index (CONTAG), shannon’s index (SHDI), shannon’s evenness index (SHEI), LPI, LSI and IJI. These indices were selected and calculated by using Fragstats v4.2.1 software^3^, and were described in Table s1 (supplementary materials). Because the significant UHI and its obvious adverse impact on human sense of comfort occurs in summer, the subsequent section of the study focused on the urban LST and associated landscape pattern factors was selected on 6 September 2020 in the hottest summertime.

## Results

### Spatial and temporal patterns of urban land use and LST

**Figure 3:**
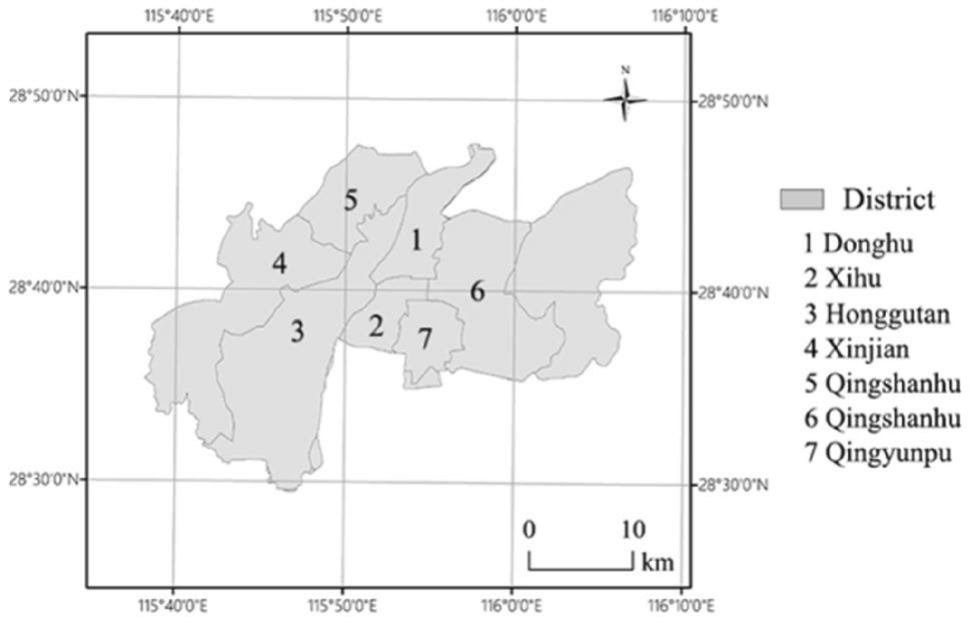
The districts map of urban Nanchang.

**Figure 4:**
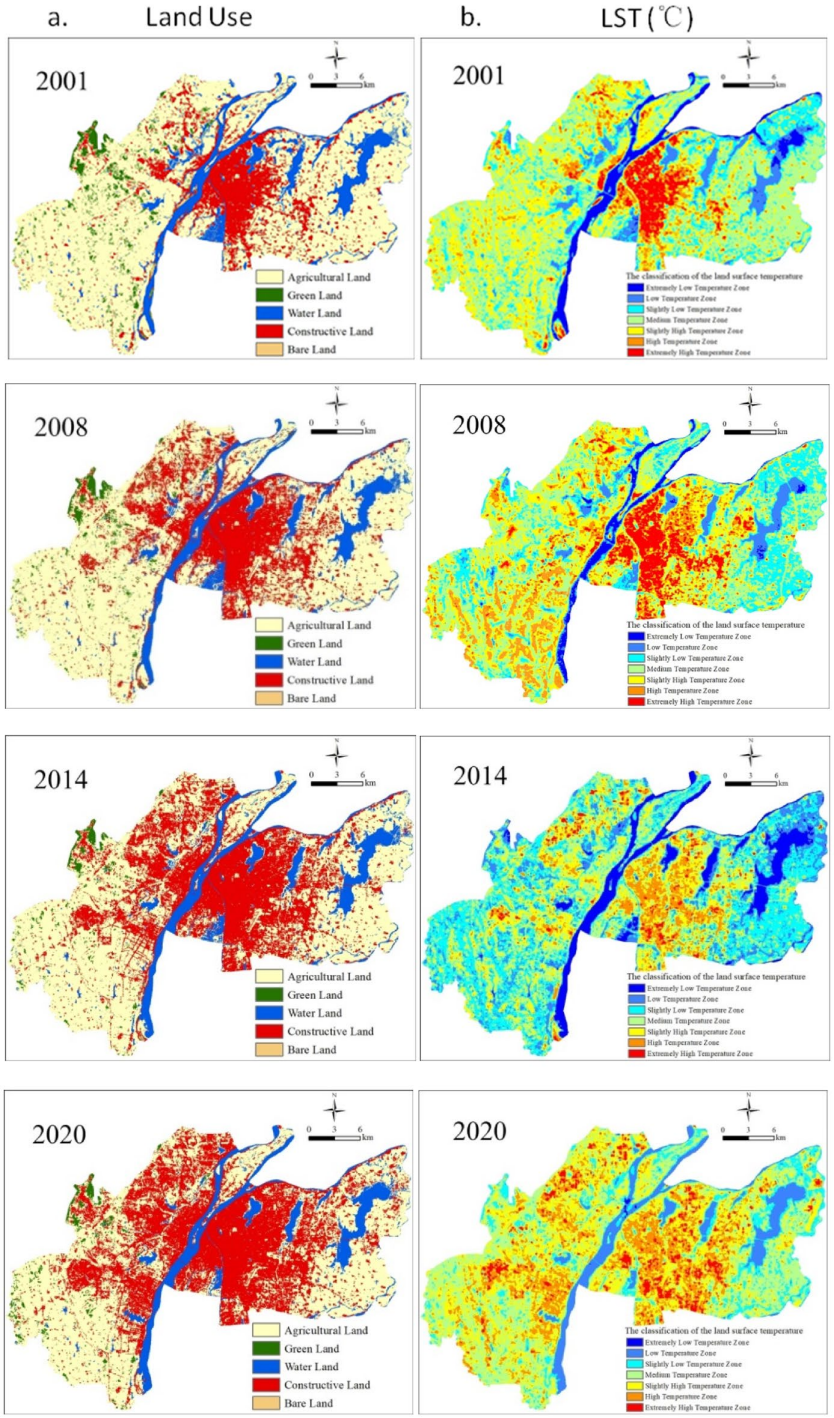
The land use maps (**a**) and LST zones (**b**) in 2001–2020.

Figures 3 and 4a showed the obvious spatial and temporal variations of areas of land use types from 2001, 2008, 2014 to 2020. Amongst them, area of CL (red) was gradually increasing in spatial pattern, contrastively, the spatial pattern of area of AL (yellow) was reductive with the year. These changes reflected the urbanization process of Nanchang in 2001–2020 (Figs. 3, 4a). In 2001, the urban CL was distributed mainly within Xihu and Donghu districts (the old urban center districts) in the east of Ganjiang River, while other districts had higher agriculture coverage rates. In 2008, Nanchang began to expand the urban area by planning of Nanchang Municipal Government, including the construction of Honggutan New District of the west of Ganjiang River, Qingyunpu District, and Qingshanhu District, the CL was scattered distribution with very few connected to each other. In 2014, continuous constructive districts were formed in six districts. Compared to 2014, in 2020, the density of CL had increased significantly and AL area had decreased obviously. GL, WL, and BL relatively little change than CL and AL. According to Figs. 3 and 4a, compared to 2001, the land use in 2020 had changed dramatically within the outer ring road of Nanchang. The urban area of Nanchang had increased from 85 km^2^ in 2001 with a population of 4,434,200 (the 2001 census) to more than 350 km^2^ in 2020 (satellite map) with a permanent population of 6,255,000.

As shown in Fig. 4b, Nanchang city had apparent hot spots in any given years, displaying that the total area of EHTZ and HTZ had amplified obviously while the MTZ and SLTZ had reduced obviously. In 2001, the UHI were assembling only in the old city center of Xihu and Donghu districts like that of CL. In 2008, the UHI downtown extended to the western where Honggutan District is located. This area is a new commercial and economic center with plenty of building beside the Ganjiang River. In 2014, the UHI expanded toward the suburban areas of each districts, and a series of discrete EHTZ and HTZ zones formed due to the development of new city areas. Compared to 2014, in 2020, hot spots had appeared obviously in the almost half of the city where was covered by higher temperature zones, reflecting further expansion of hot spots.

Average LST had a similar spatial pattern with Land use (Fig. 4a). i.e., the distribution of EHTZ and HTZ (red zone) were consistent with that of CL (red); the distribution of SHTZ (yellow) responded to that of AL and BL with higher vegetation coverage rates; the distributions of MTZ and SLTZ (nattier blue) were corresponding with that of GL; and the distribution of LTZ and ELTZ (deep blue) responded to that of WL with large heat capacity and low thermal conductivity^21,36^. In 2020, obvious hot spots had appeared in the almost half of the city due to large contiguous tracts of CL. These results showed the expansion of urban LST is basically consistent with the expansion pattern of urban land use types.

**Figure 5:**
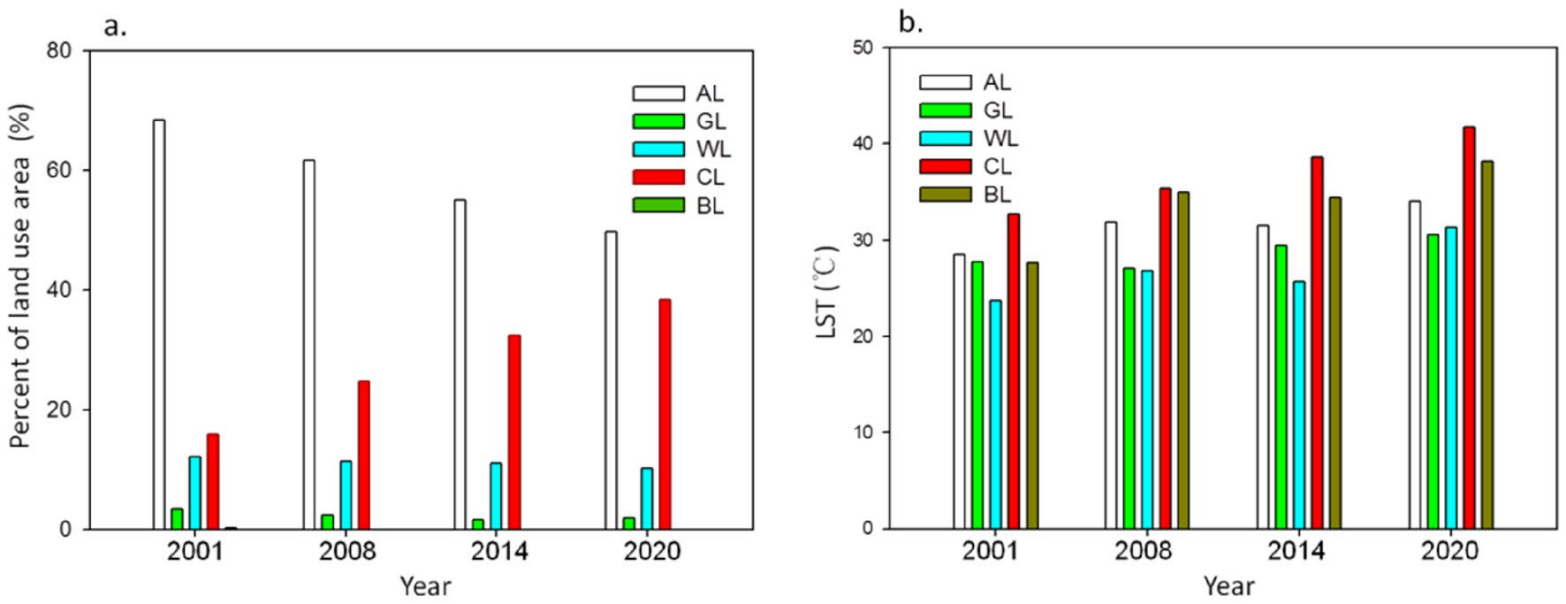
The area percent (**a**) and LST (**b**) of each land use types with the years.

The variations of areas of land uses were also presented in Fig. 5a and Table s2. Area of CL accounted for 15.93% in 2001 to 38.32% in 2020, rapidly increased by 22.5%, inversely, AL areas accounted for 68.44% to 49.69% from 2001 to 2020 and a sharp decrease by 19%, and WL areas decreased by 2% from 12.06% to 10.15%. The LST features of land use types are achieved attribute to different absorbing and reflection rate of the solar radiation by land coverage^14,37^. According to Fig. 5b, LST of land uses significantly varied with the years. These changes indicated an adverse effect on the thermal environment through the urbanization process from 2001 to 2020. The LST of CL was highest and increased with the years because of amplified CL area, showing that CL was the most significant factor contributing to LST and thereby heat environment effect^9,27^. LST of GL and WL showed significantly lower than that of CL and BL. The sequence of average LST rankings from the high to the low were CL, BL, AL, GL and WL. The overall average LST was hotter and hotter with the years when massive AL were replaced by CL (Figs. 4a,b and 5a,b).

### The relation between grade of LST and area percent of land use

**Figure 6:**
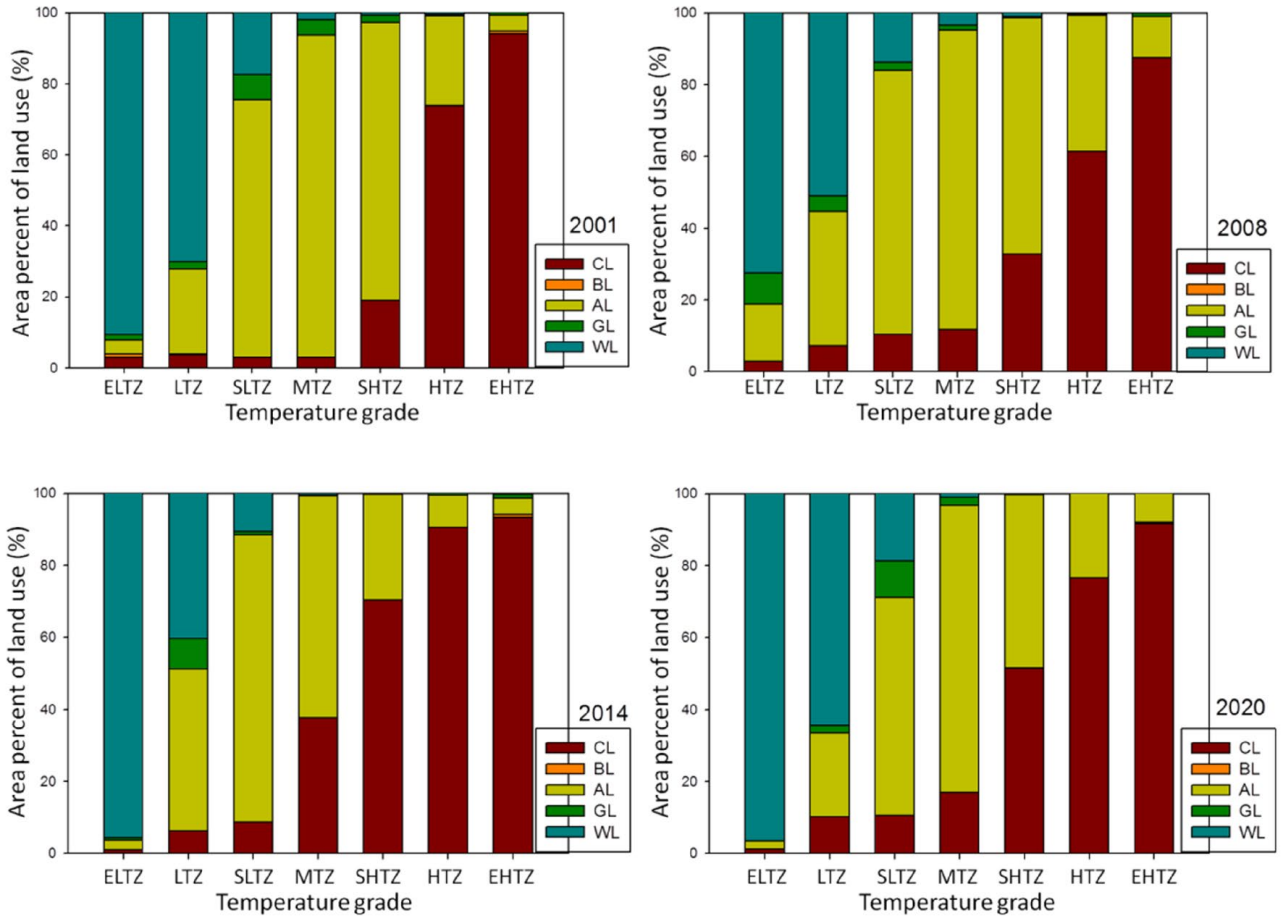
The area percent of each land use types in different LST zones in 2001–2020.

The area ratios of land uses in each LST zones were calculated by the spatial statistic function of ArcGIS (Fig. 6). Combined Table s3 (supplementary materials), the results showed the temperature zones were significantly variation with the years. The EHTZ, HTZ and SHTZ increased whereas the MTZ, SLTZ and LTZ decreased with the years. The maximum change in ratios happened in the MTZ and HTZ, EHTZ, showing area percent of CL increased whereas that of AL decreased. And the ratio changes of SLTZ (− 4.77%), MTZ (− 6.95%), SHTZ (6.17%), HTZ (5.28%), and EHTZ (3.86%) were more significant between 2014 and 2020 than those between 2001 and 2008 (2.67%, − 4.84%, − 3.31%, 1.65%, 0.93%, respectively) (Table s3), indicating that heat intensity increased rapidly between 2014 and 2020. The area ratios of land uses in different temperature zones significantly varied, however, the result of each year showed the same features, i.e., CL was the largest portion of EHTZ and HTZ showing CL contributed the most to heat effect^38^; AL was the largest part in SHTZ, MTZ and SLTZ; and WL was the largest part in LTZ and ELTZ, showing WL contributed the most to cooling effect; GL was small part in LTZ and SLTZ. Area percent of BL was small and can’t be displayed.

### Quantitative analysis of LST and landscape composition

**Figure 7:**
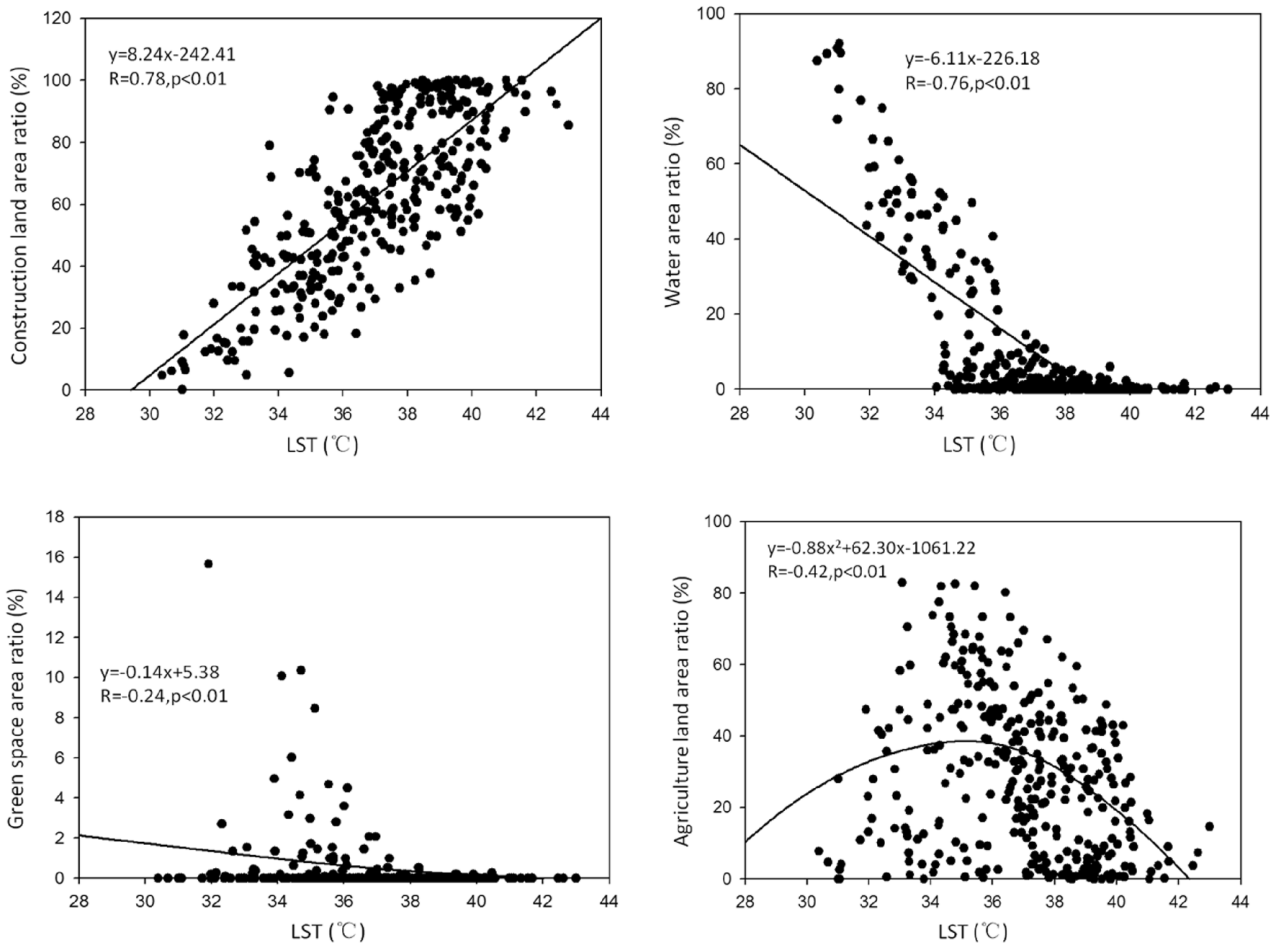
The quantitative relationship between LST and area ratios of land uses.

According to Fig. 7, there were a good linear fitting relationship (*p* < 0.01) between LST and the area ratios of CL, WL, GL and AL, indicating the four land use types can better explain the changes of LST. The linear fitting of LST and area ratio of CL was the strongest and had a significant positive correlation (R = 0.78), indicating the larger proportion of CL and the higher LST. There were a significant negative correlation between LST and AL, GL and WL, indicating the higher the proportion of AL, GL and WL can better mitigate the region's high temperature, especially WL had a best cooling effect (R = − 0.76) for this city.

In order to obtain a more accurate model of the relationship between LST and the overall land use, multiple regression analysis was carried out between LST and area percent of land use^15^, and the contribution value of different land uses to LST was obtained. The prediction model was shown in Eq. (6).

6 $${\text{T }} = { 39}.{95}0 \, + \, 0.0{\text{16CL }} - \, 0.{19}0{\text{GL }} - \, 0.0{\text{51AL }} - \, 0.{1}0{\text{7WL }} + \, 0.00{\text{1BL}}$$

where T stands for LST. The coefficient of the model was the coefficient of the variable in Table 3. The model fitted well (R = 0.87), can be used to predict the LST and adjust the area of various land use types to achieve the optimal land use planning of urban^15^.

**Table 3:** The multivariate linear regression of LST and area ratios of land uses.

Model	Coefficients	Std. Error	t	Sig	R
K_0_	39.950	0.782	51.087	0.000	0.87
AL	− 0.051	0.017	− 3.000	0.004	
GL	− 0.190	0.050	− 3.800	0.000	
WL	− 0.107	0.017	− 6.294	0.000	
BL	0.001	2.621	0.000	0.762	
CL	0.016	0.018	0.889	0.004	

### Quantitative relation of LST and patch type indices of land use

There were significant correlation between LST and six landscape pattern indices of CL (*p* < 0.01) (Table 4 and Fig. s1), with significant positive correlations with PLAND (y = 8.20x − 240.98, R = 0.78), LPI (y = 8.82x − 268.20, R = 0.73) and MNN (y = 0.34x − 8.57, R = 0.44), and negative correlations with NP (y = − 0.64x + 28.22, R = − 0.45), LSI (y = − 0.16x + 9.30, R = − 0.35) and IJI (y = − 4.33x + 194.35, R = − 0.34). These results showed that large PLAND and LPI values indicated the larger the area ratio and the more concentrated distribution of CL generated the higher LST, due to CL generally made of concrete and other heat-absorbing materials^16,23^. Large NP, IJI and LSI intended the more fragment, dispersed and complex shape distribution of CL help to alleviate the thermal environment due to massive surface contact with the surroundings.

**Table 4:** The correlation between LST and patch type metrics of land use.

Temperature	PLAND	NP	LPI	LSI	MNN	IJI
LST_CL_	0.78**	− 0.45**	0.73**	− 0.35**	0.44**	− 0.34**
LST_WL_	− 0.78**	− 0.27*	− 0.75**	− 0.33**	0.16*	0.32*
LST_AL_	− 0.63**	− 0.35**	− 0.64**	− 0.32*	0.20*	− 0.49**
LST_GL_	− 0.42**	− 0.28*	− 0.38**	− 0.32*	0.21	0.31*

**Significant at *p* < 0.01 (bidirectional); *Significant at *p* < 0.05 (bidirectional).

Although all the metrics of WL had a significant correlation with LST (*p* < 0.05) (Table 4 and Fig. s2), PLAND (y = − 7.92x + 301.38, R = − 0.78) and LPI (y = − 7.05x + 268.45, R = − 0.75) had the most strong negative correlation with LST (*p* < 0.01), meaning the larger the area ratio and the more concentrated distribution of water body, the lower LST. NP (y = − 0.09x^2^ + 6.37x − 109.27) and LSI (y = − 0.03x^2^ + 2.05x − 33.84) had significant fitting with LST, showing the more number and complex shape of WL, the more effectively reduce LST. In addition, high temperature vs a very low area proportion (even 0%) of WL in Fig. s2 indicated that temperature surrounding water body was relatively high and water body had a cooling effect. IJI (y = 0.32x^2^ − 19.56x + 354.85, R = 0.32) had positive correlations with LST, indicating the dispersed distribution of WL can weaken its cooling effect to some extent.

LST had negative significant correlation with five metrics of AL (*p* < 0.05) except MNN (Table 4 and Fig. s3), showing that AL can reduce heat effect. However, the correlation coefficients were smaller between PLAND (y = − 5.49x + 235.74), LPI (y = − 5.24x + 232.99) of AL and LST than those of WL and LST, indicating AL was weaker than WL to relieve heat effect. This can also be confirmed by the majority of AL in SHTZ, MTZ and SLTZ (Fig. 6).

The relationship between LST and patch type indices of GL was similar to that between LST and patch type indices of AL (Table 4 and Fig. s4). In other words, the cooling effect of a large area of GL was stronger than that of multiple small areas of equal area, and the more concentrated distribution and more complex shapes of GL patches were better the cooling effect^3,16^.

### Quantitative correlation between LST and landscape level indices

Table 5 showed the correlation between LST and landscape pattern metrics at landscape level. LST was significantly correlated with seven landscape level indices (*p* < 0.01), amongst them, LST was significantly negatively correlated with PD, LSI, IJI, SHEI and SHDI, in contrast to LPI, CONTAG and AI. In other words, large PD, SHEI and SHDI values indicated the richer diversity of landscape corresponded to the lower LST; The large the LSI values showed the more complex shape characteristic, the lower LST; The high LPI, CONTAG and AI values showed the lower fragmentation degree of landscapes were consistent with the higher LST. When considering the effect of mixed landscape configurations, the fragmentation degree, shape characteristic and diversity were more important to alleviate LST than other spatial arrangement features. It was noticed some studies also showed a significant effect of landscape metrics in explaining the thermal environment intensity based on fragmentation, shape, connectivity and patch size^39,40^, for instance, relatively large water body presented a higher cooling effect than equally distributed small waterbodies^41,42^, while the fragmentations of construction land were conducive to reduce the thermal environment^23^, these results were obtained from individual patch characteristic of land cover (patch level), not from the overall characteristics of different land cover (landscape level).

**Table 5:** The correlation between LST and landscape pattern indices at landscape level.

Temperature	PD	LPI	LSI	CONTAG	IJI	AI	SHDI	SHEI
LST	− 0.33**	0.52**	− 0.42**	0.41**	− 0.45**	0.44**	− 0.60**	− 0.45**

**Significant at *p* < 0.01 (bidirectional).

## Discussion

There were strong correlations between LST and urban land uses^43^. With the rapid urbanization development of Nanchang in the past 20 years in this study, urban heat spots had covered most areas of the city due to increasing in the coverage of impervious surfaces and to decreasing in natural surface, consequently, high heat capacity and less evapotranspiration from surface had existed^27,44^. Similarly, construction land corresponded to extremely high temperature zone (EHTZ) and high temperature zone (HTZ) in this study. On the other hand, water body was consistent with extremely low temperature zone (ELTZ) and low temperature zone (LTZ). Hence, in landscape planning and design, the landscape of CL should be dispersed as far as possible by the landscape of GL or WL, which can effectively alleviate urban thermal environment. The conclusion was also consistent with the overall characteristics of different land cover (landscape level) in affecting LST.

Some studies had shown a significant effect of landscape pattern metrics on LST^39,40^. The area proportion of land use was the most important indicator of the contribution on LST. The larger the area ratio and the more concentrated distribution of CL generated the higher LST, whereas the more fragment, dispersed and complex shape distribution of CL help to alleviate the thermal environment. WL, AL and GL Landscapes can be regarded as ecological landscapes, indicating their cooling effects for the entire city^44,45^. The area percentage of ecological landscapes had the most obvious negative correlation with LST, followed by the fragmentation and the shape indices, showing that compositions and configuration features of landscapes were all important in affecting LST^30,46^. The multiple regression analysis was carried out between LST and area percents of land uses^15^, and the contribution value (0.87) of different land use areas to LST can be obtained. However, the contributions of many landscape metrics to the heating/cooling effect are still unclear^14,40^. Therefore, a careful planning of land use is important based on landscape configurations in order to maximise the benefit of the policy strategy.

## Conclusions

1. Heat environment of Nanchang had become more worse with continuous expansion and enhancement of urbanization area in the past 20 years. In 2001, hot spots were mainly concentrated on the old downtown. In 2020, with comprehensive expansion of construction land, hot spots had gradually occupied a dominant position all around the city.
2. LST of different land uses were significantly different, and the order was: CL > BL > AL > GL > WL. CL contributed the main heating effect. GL has a certain transpiration and shading effect, but GL occupied a relatively small area in this study. WL formed sheet surfaces and continuous bands and had a certain land area, therefore, WL had a better cooling effect.
3. The relations of LST and patch indices of land uses showed that the larger area of GL, AL, WL, the smaller landscape fragmentation degree, the more concentrated the distribution, and the more complex shape responded to the lower LST, in contrast to those of CL.
4. The relation between LST and landscape level indices showed that LST was negatively correlated with PD, LSI, IJI, SHEI and SHDI, and positively correlated with CONTAG, LPI and AI. Hence, in order to reduce the surface temperature in the region, a variety of different types of landscapes should be set as far as possible, and the degree of aggregation between different landscapes should be low.

### Supplementary Information

Supplementary Information.

## Data Availability

The datasets generated during and/or analyzed during the current study are available from the corresponding author on reasonable request.
