# Supplementary material for: Effects of landscape pattern on land surface temperature in Nanchang, China
Source: Sci Rep. 2024 Feb 15;14:3832. doi: 10.1038/s41598-024-54046-4 (PMC10869699; doi:10.1038/s41598-024-54046-4)
Supplement: Supplementary file 1 — Supplementary Information. [file 41598_2024_54046_MOESM1_ESM.docx]

**Supplementary materials: Table s1- s3, Fig.s1-Fig.s5**

Table s1 Landscape pattern indices and their ecological meaning (Mcgarigal et al., 2012)

| Metric (class-level) | Description |
| --- | --- |
| Percent of Landscape (PLAND) | Proportion of landscape type to total landscape (%) |
| Landscape Shape Index (LSI) | Perimeter of patch divided by perimeter of circle with the same area as the patch |
| Number of Patches (NP) | Count of total number of patches |
| Largest Patch Index (LPI) | Area ratio of largest patch in landscape to study area (%). Value near 0 indicates that area of largest patch is small. Value of 100 means entire landscape composed of only one type of patch. |
| Mean Euclidean nearest-neighbor distance (MNN) | Mean distance to nearest neighboring patch of landscape type based on edge-to-edge distance (m). |
| Interspersion and Juxtaposition Index (IJI) | The degree of mixed distribution of each patch type, when each patch is adjacent to only one other type of patch, IJI tended to 0; When the adjacency probability of each patch is the same as that of all other plaque types, IJI tend to 100. |
| Patch Density (PD) | The number of patches within 100ha. The higher the patch density, the more serious the landscape fragmentation degree. |
| Contagion (CONTAG) | The aggregation degree of different patch types, when the landscape is composed of many small patches, the spread value is lower, and when the dominant patch types in the landscape have better connectivity, the value is higher. |
| Aggregation index (AI) | AI equals the number of like adjacencies involving the corresponding class. AI equals 0 when the focal patch type is maximally disaggregated (i.e. when there are no like adjacencies). It increases as the focal patch type is increasingly aggregated and equals 100 when the patch type is maximally aggregated into a single, compact patch. |
| Shannon’s Diversity Index (SHDI) | The complexity of the system structure reflects the change of landscape elements and the proportion of each landscape element. SHDI=0 indicates that the landscape is composed of only one patch. The increase of SHDI shows the increase or the balanced division of patch types in the landscape. |
| Shannon’s Evenness Index (SHEI) | The uniformity of spatial distribution of different landscape types is between 0 and 1. SHEI=0 indicates that the landscape is composed of a patch with no diversity. SHEI=1 indicate that the patch types are evenly distributed and has the maximum diversity. |

Mcgarigal, K., Cushaman, S. A., Ene, E. Fragstats v4: spatial pattern analysis program for categorical and continuous maps. Computer software program produced by the authors at the University of Massachusetts, Amherst, 15(2012).

http://www.umass.edu/landeco/research/fragstats/fragstats.html.

Table s2 Percentage of each land use in 2001-2020

|  | AL | GL | WL | CL | BL |
| --- | --- | --- | --- | --- | --- |
| 2001 | 68.44% | 3.44% | 12.06% | 15.93% | 0.14% |
| 2008 | 61.71% | 2.26% | 11.30% | 24.72% | 0.01% |
| 2014 | 54.99% | 1.53% | 11.00% | 32.47% | 0.01% |
| 2020 | 49.69% | 1.81% | 10.15% | 38.32% | 0.03% |

Table s3 The area ratio of each temperature zone (%)

| Temperature Grade | 2001 | 2008 | 2014 | 2020 | 2001-2008 | 2008-2014 | 2014-2020 |
| --- | --- | --- | --- | --- | --- | --- | --- |
| ELTZ | 4.39% | 4.51% | 4.21% | 0.77% | 0.12% | -0.30% | -3.44% |
| LTZ | 8.01% | 9.44% | 12.64% | 12.49% | 1.43% | 3.20% | -0.15% |
| SLTZ | 16.19% | 17.19% | 19.86% | 15.09% | 1.00% | 2.67% | -4.77% |
| MTZ | 40.17% | 31.43% | 26.59% | 19.64% | -8.74% | -4.84% | -6.95% |
| SHTZ | 22.59% | 22.41% | 19.10% | 25.27% | -0.18% | -3.31% | 6.17% |
| HTZ | 7.39% | 11.39% | 13.04% | 18.32% | 4.00% | 1.65% | 5.28% |
| EHTZ | 1.26% | 3.63% | 4.56% | 8.42% | 2.37% | 0.93% | 3.86% |

**Fig.s1：Construction land**


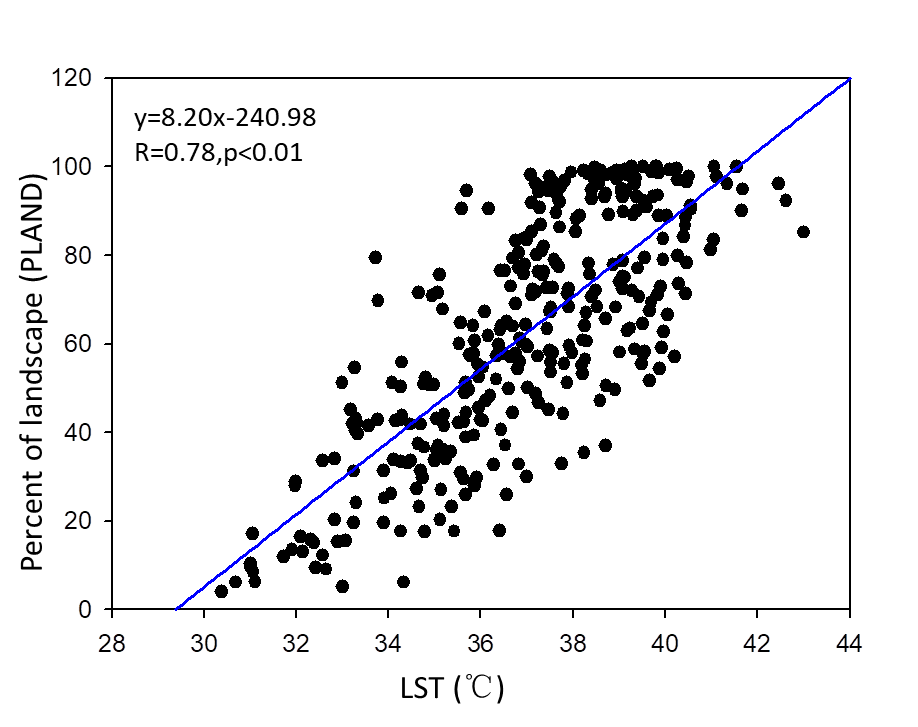

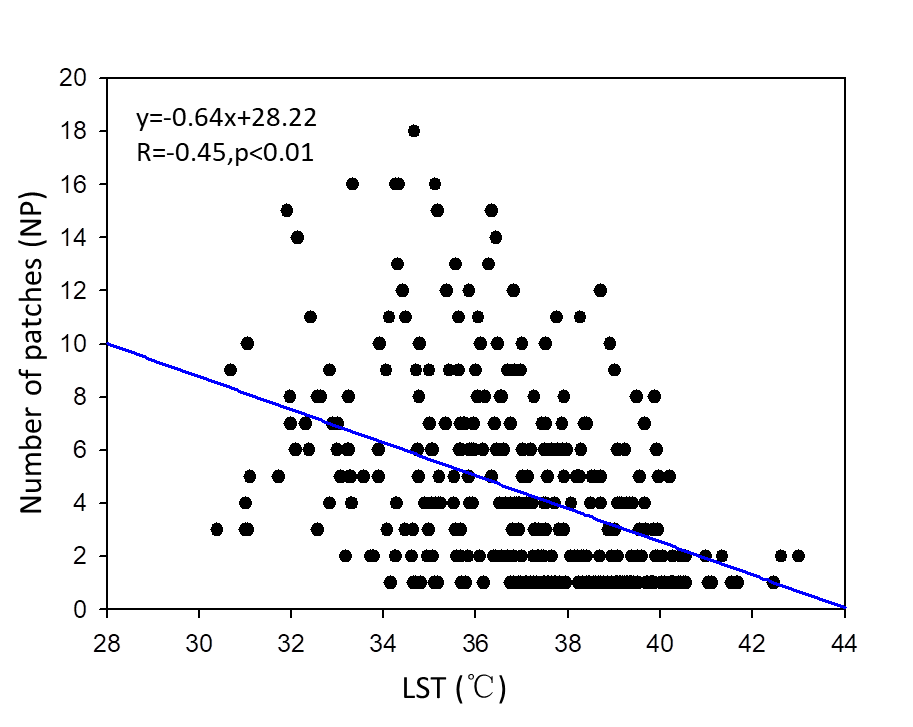


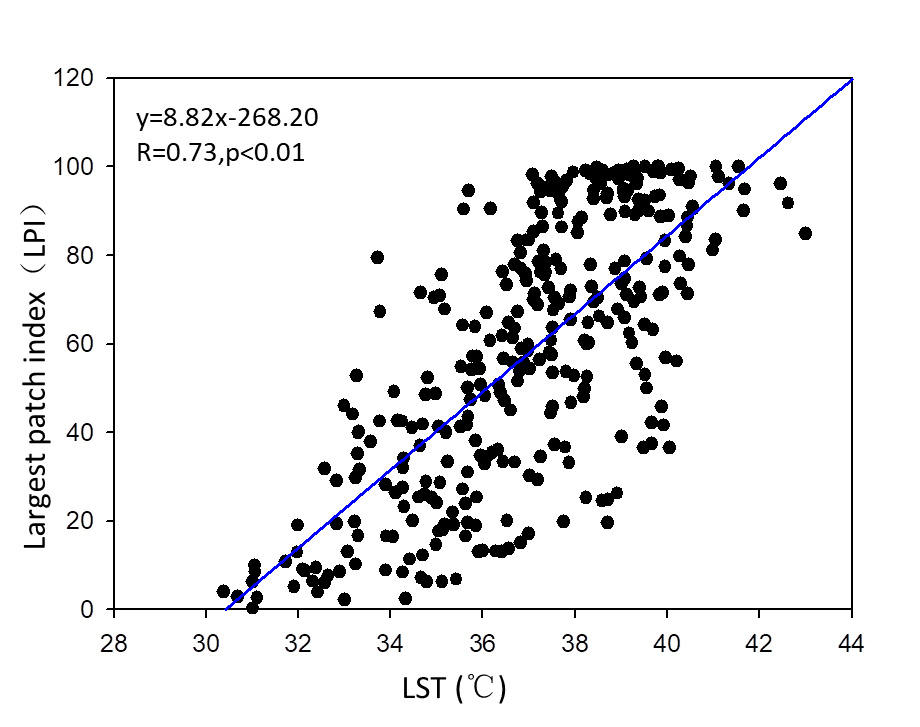

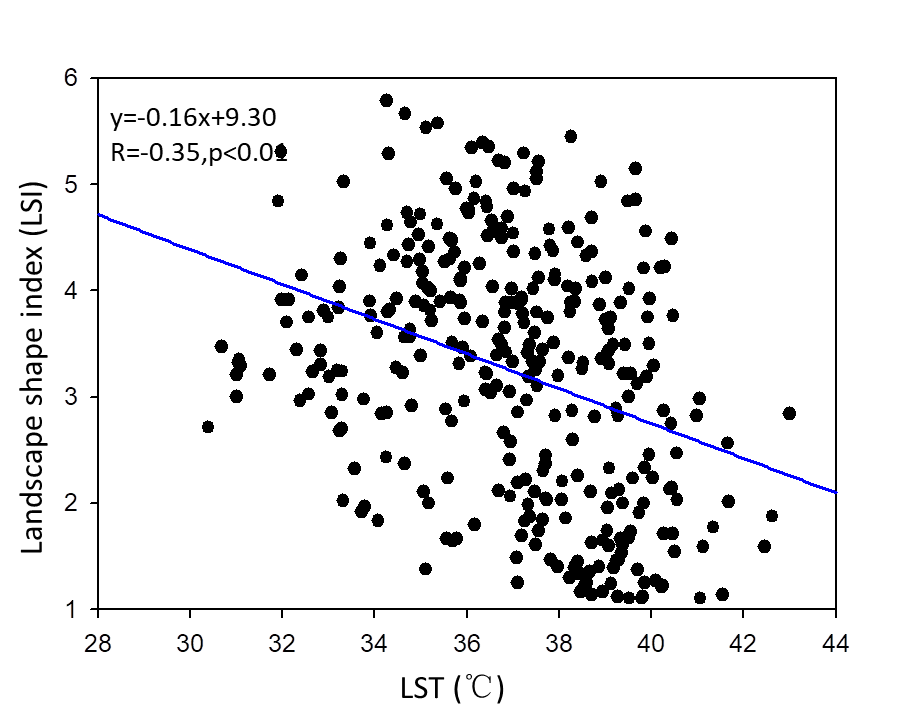


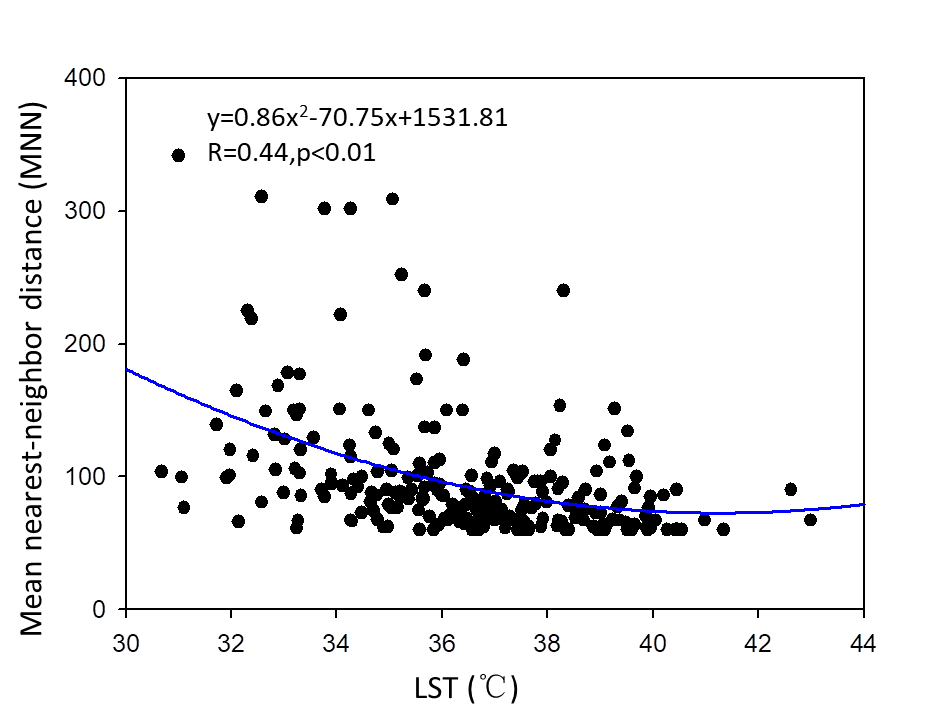

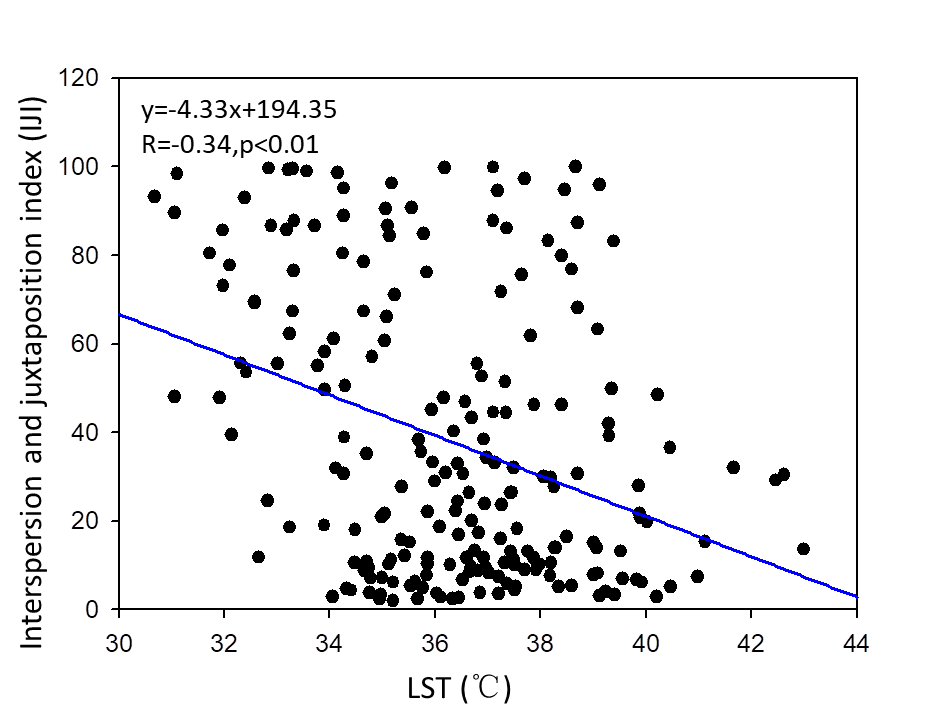


Fig. s1 The correlation between mean LST and patch type indices of CL

**Fig.s2：**Water body


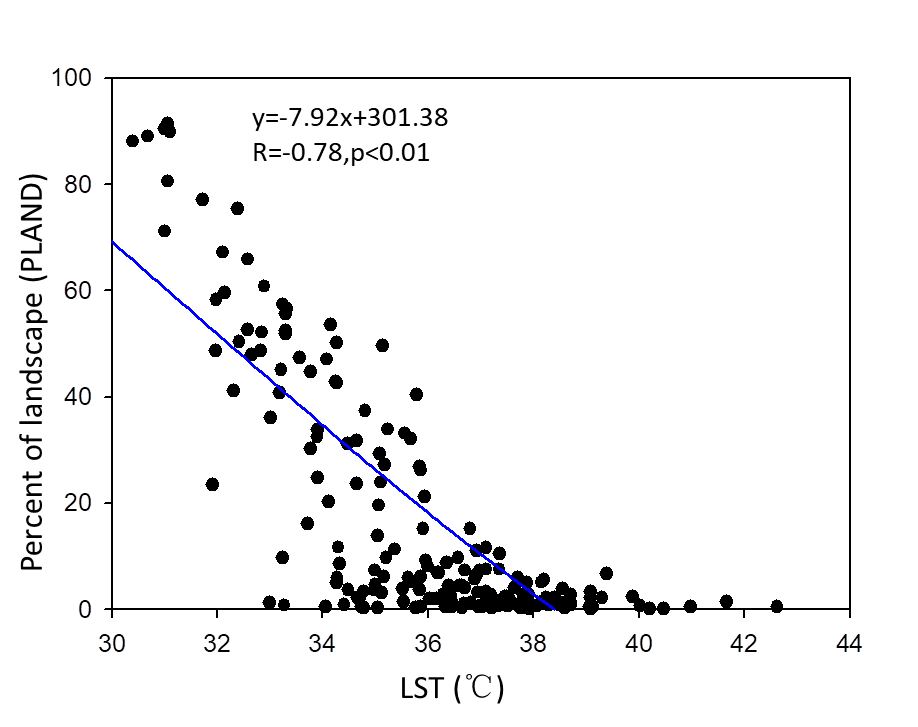

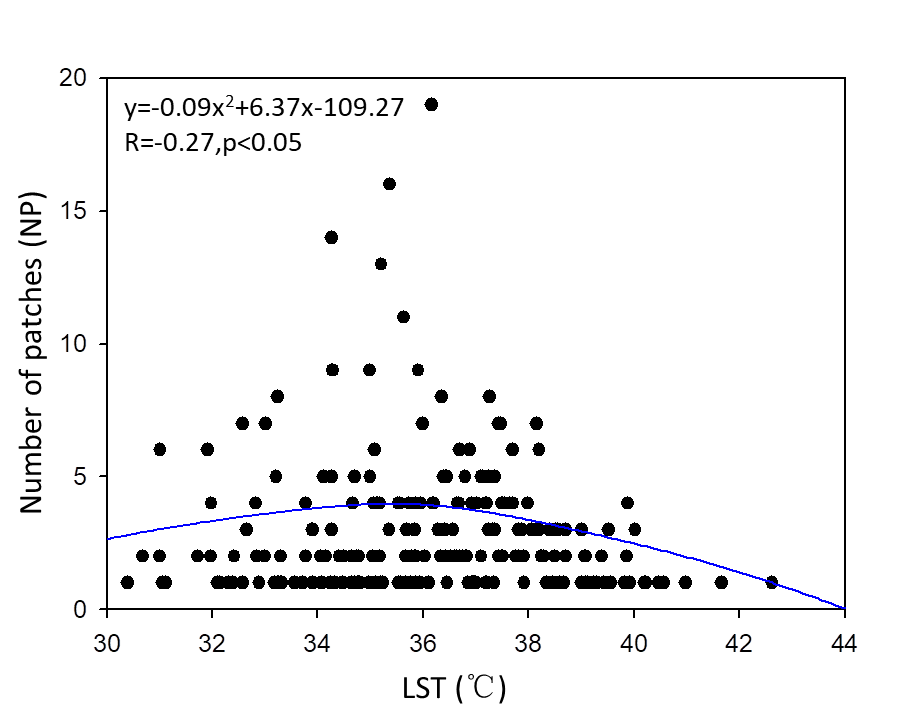


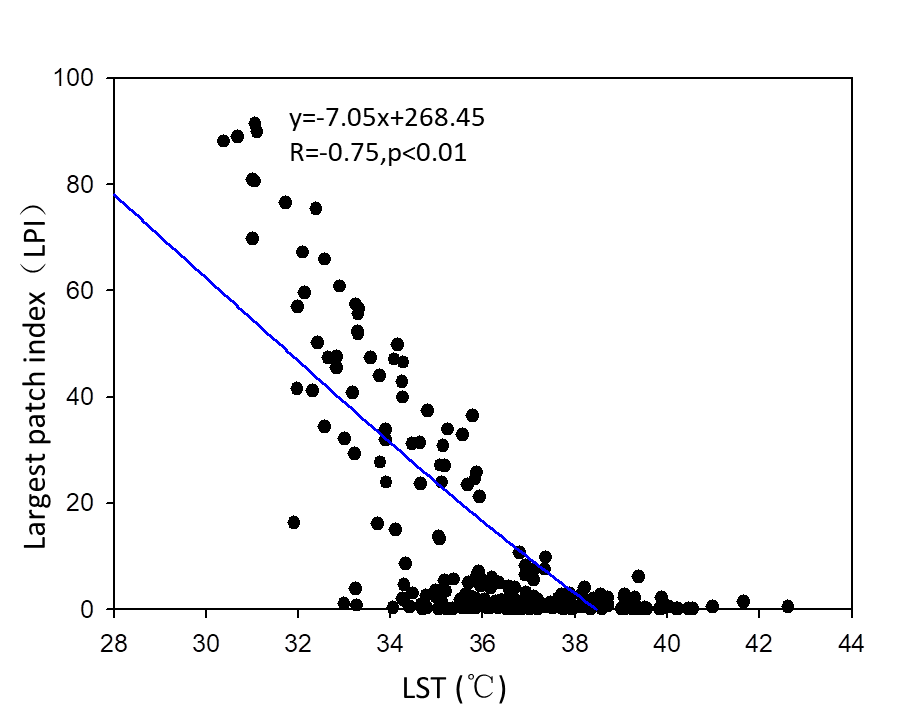

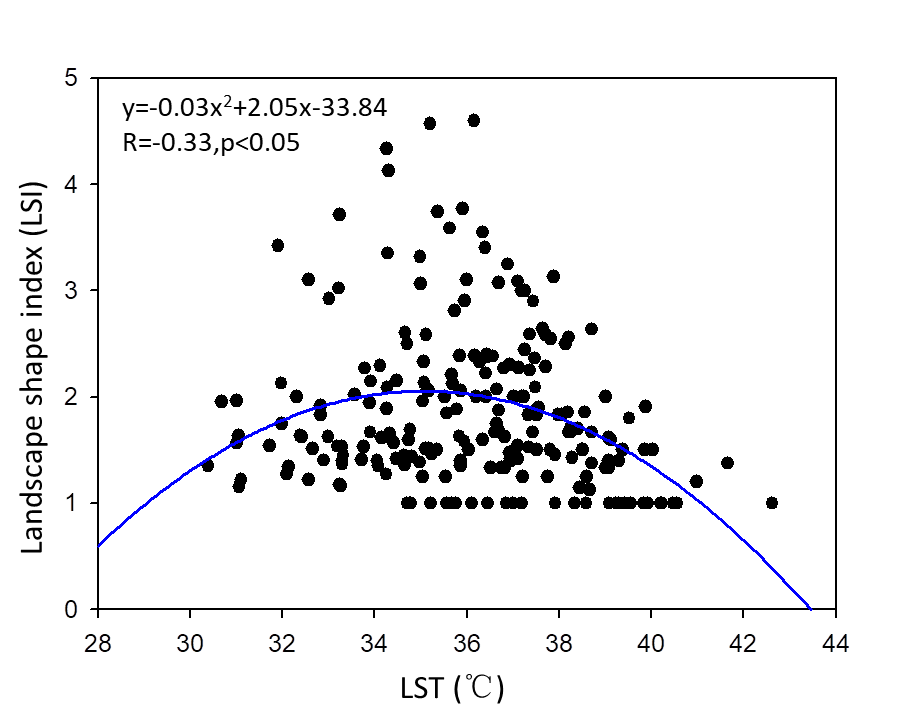


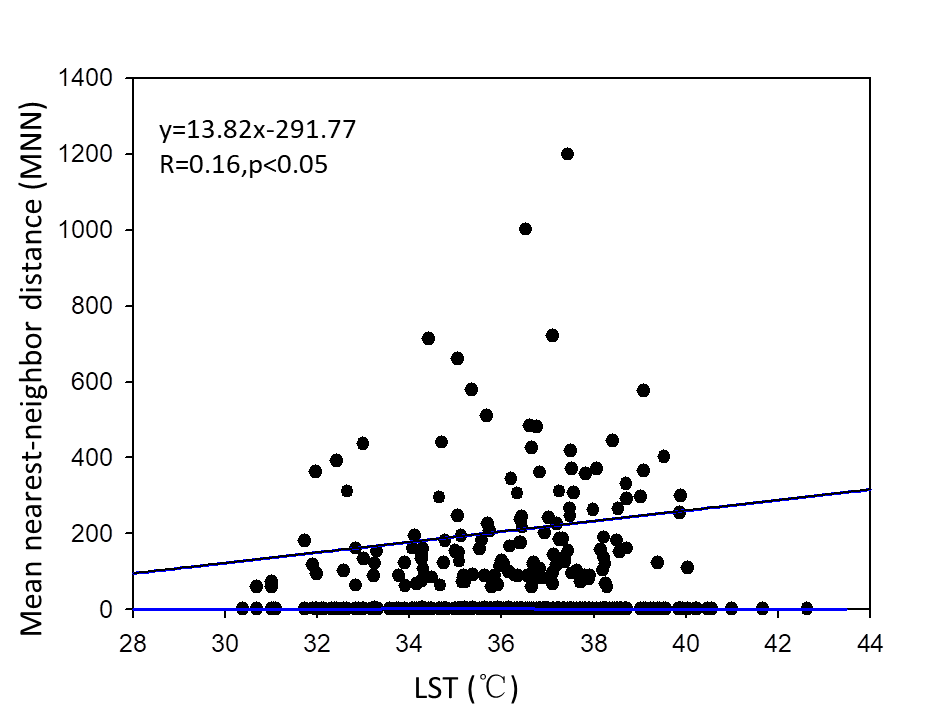

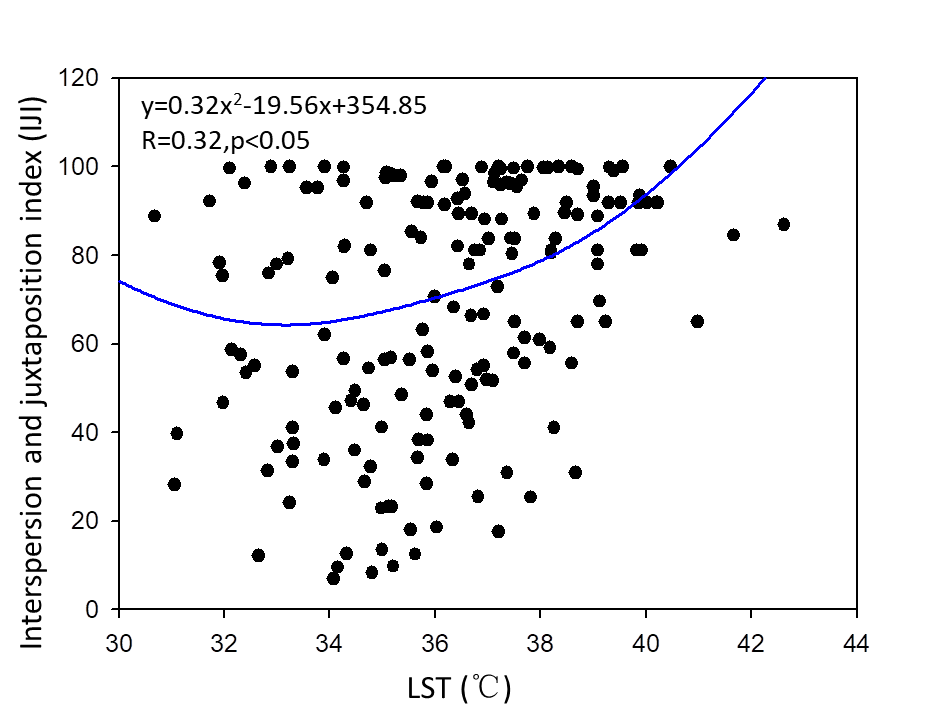


Fig. s2 The correlation between mean LST and patch type indices of WL

**Fig.s3：**Agricultural land


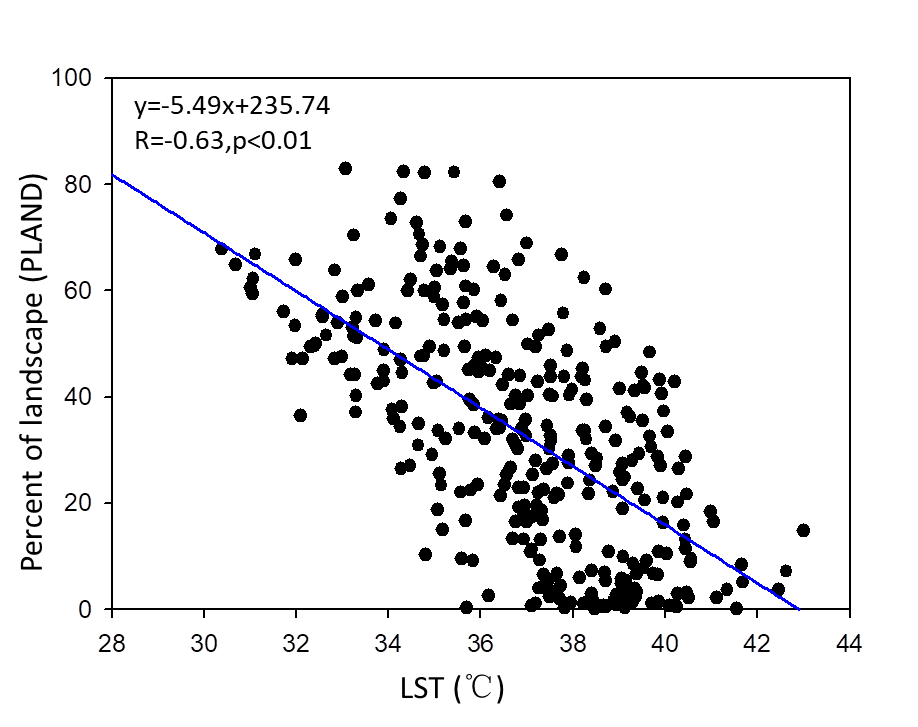

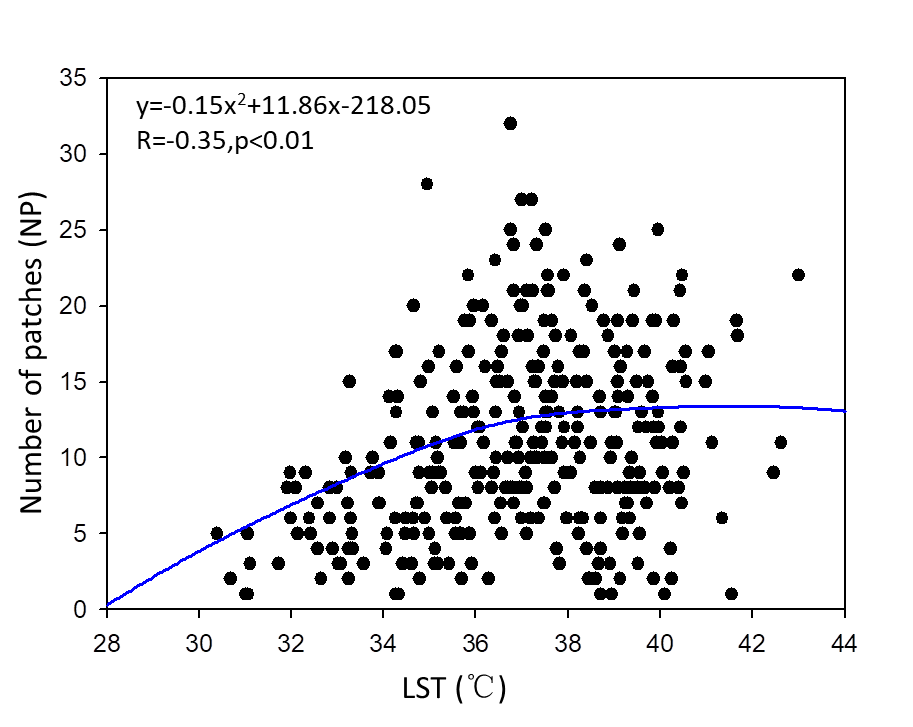


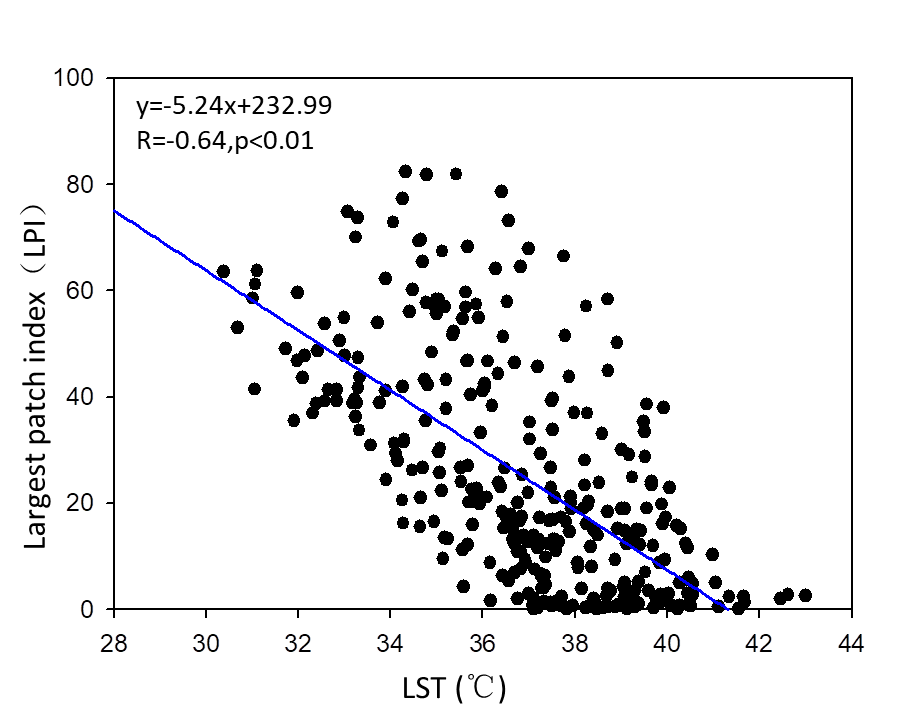

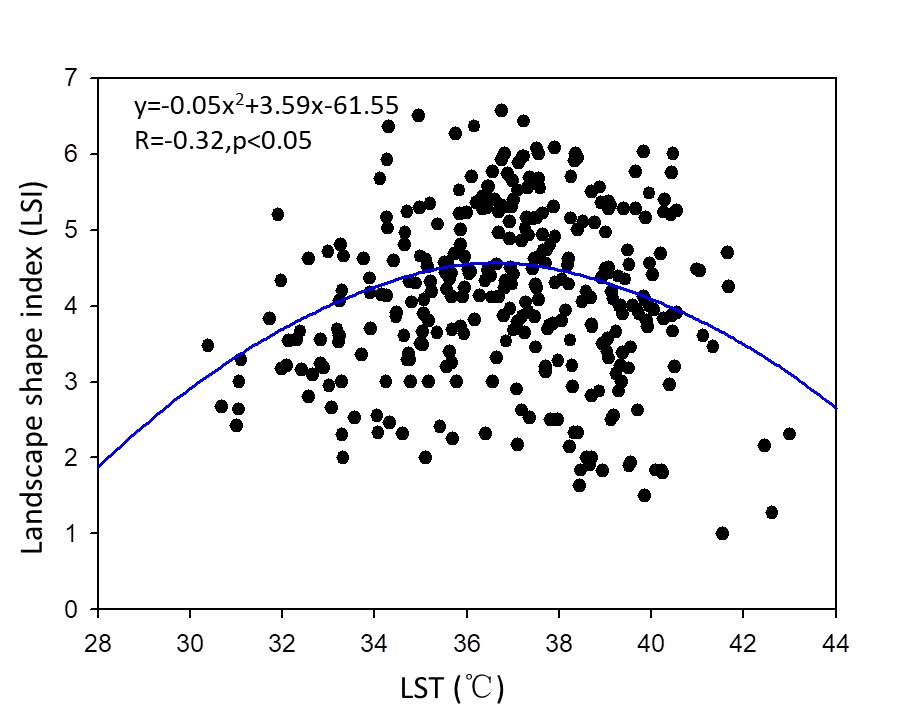


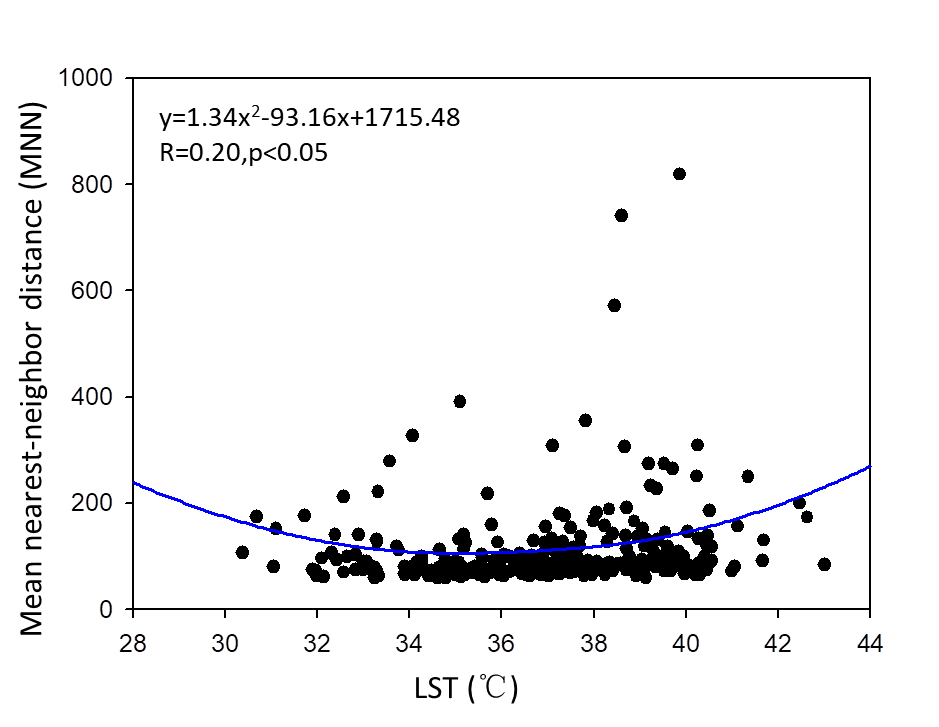

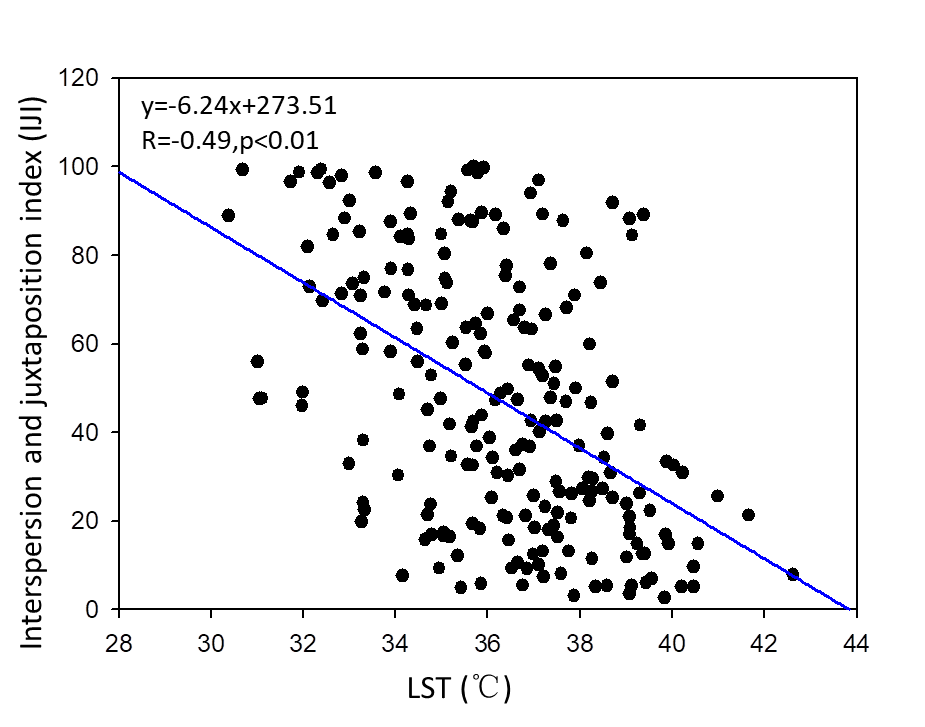


Fig.s3 The correlation between mean LST and patch type indices of AL

**Fig.s4：**Green Land


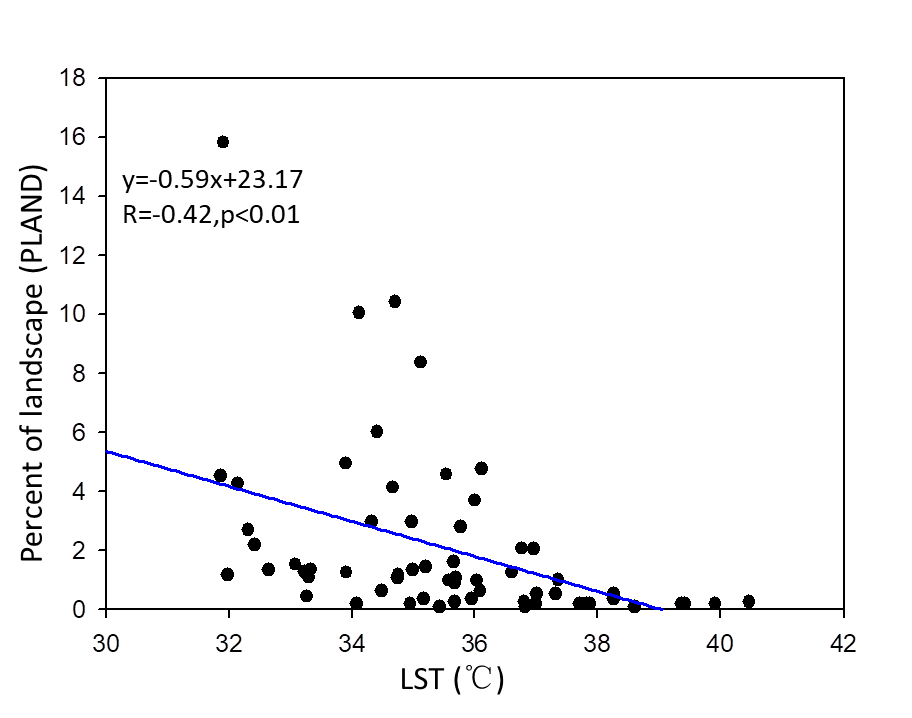

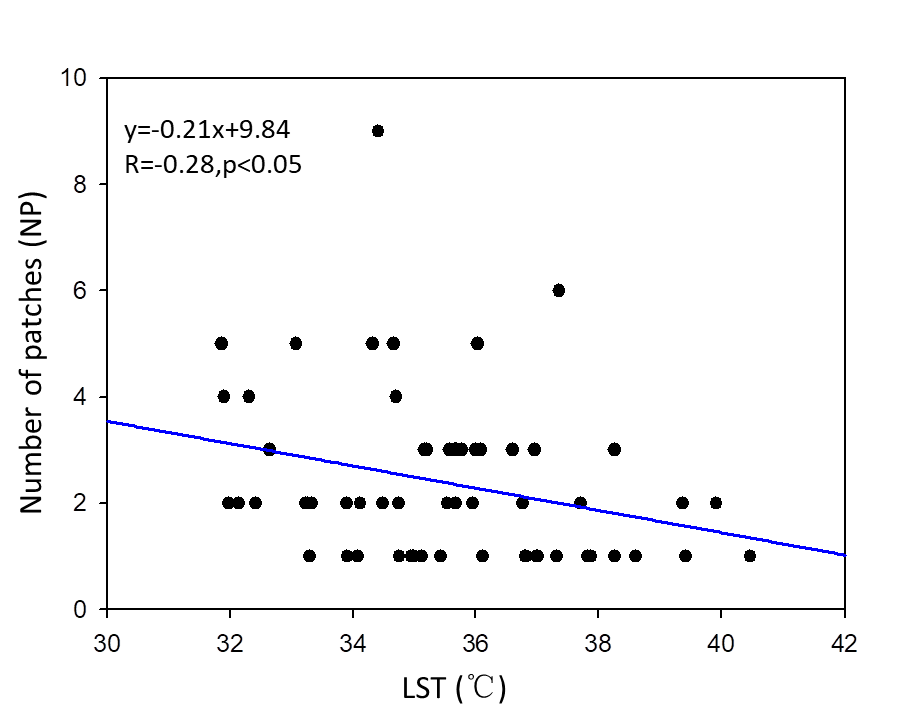


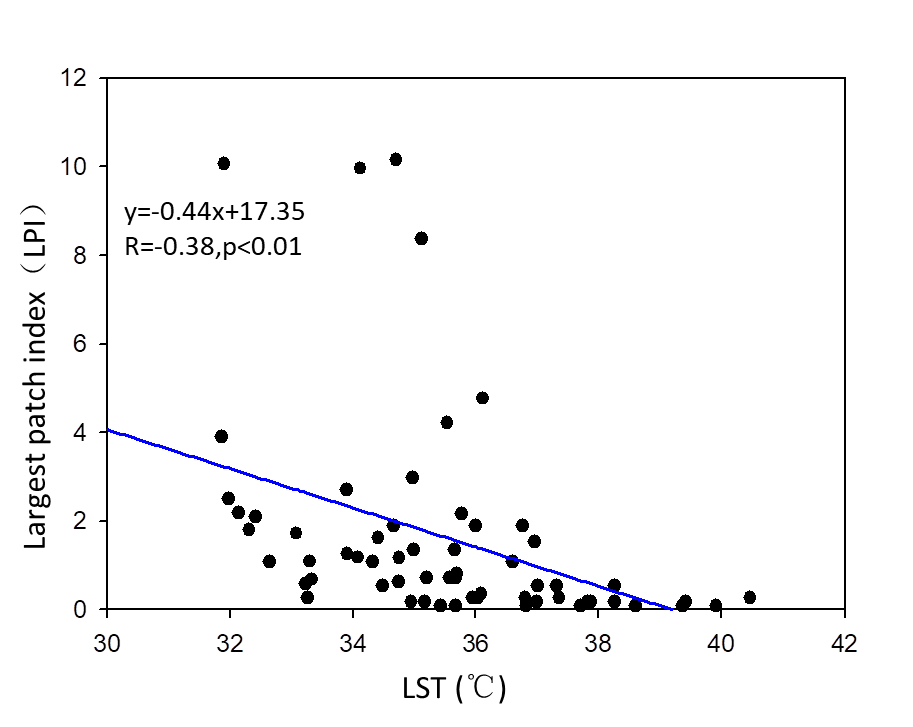

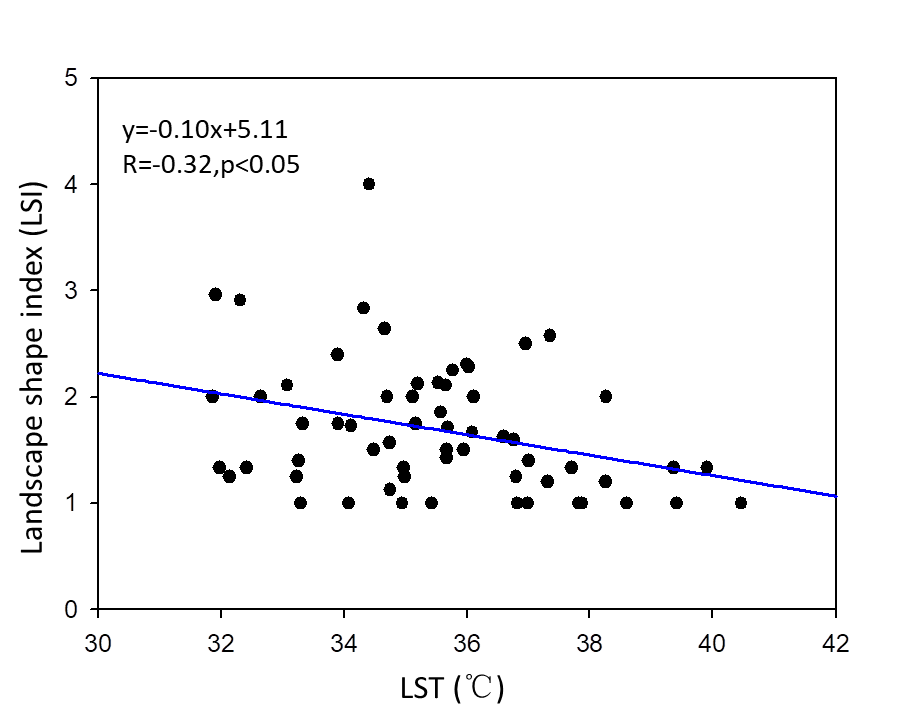


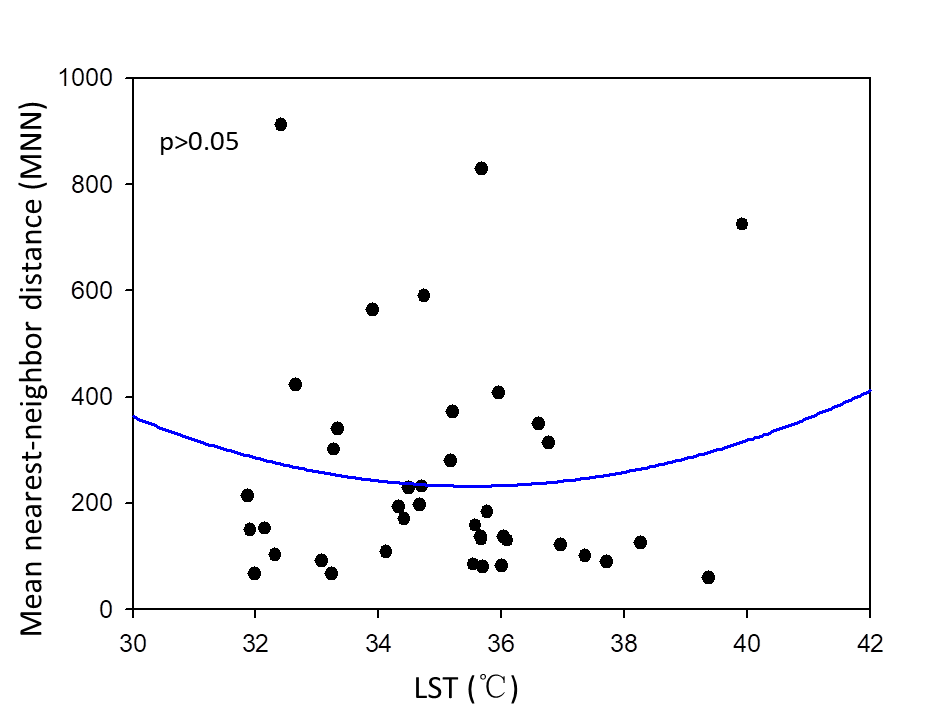

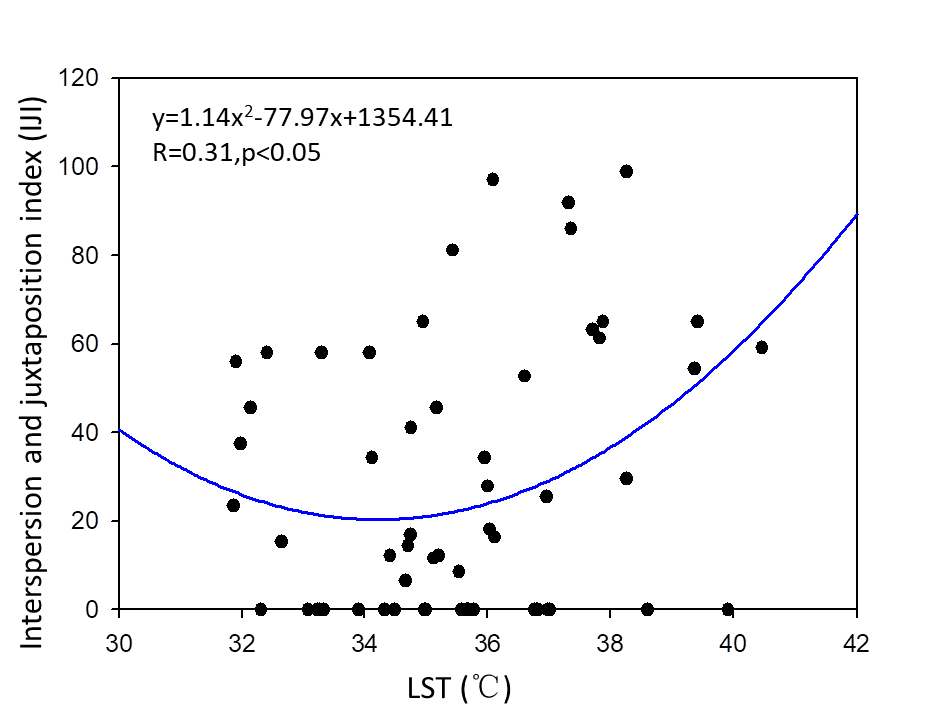


Fig. s4 The correlation between mean LST and patch type indices of AL

**Fig.s5：The relationship between LST and the different landscape pattern indexes in landscape levels**


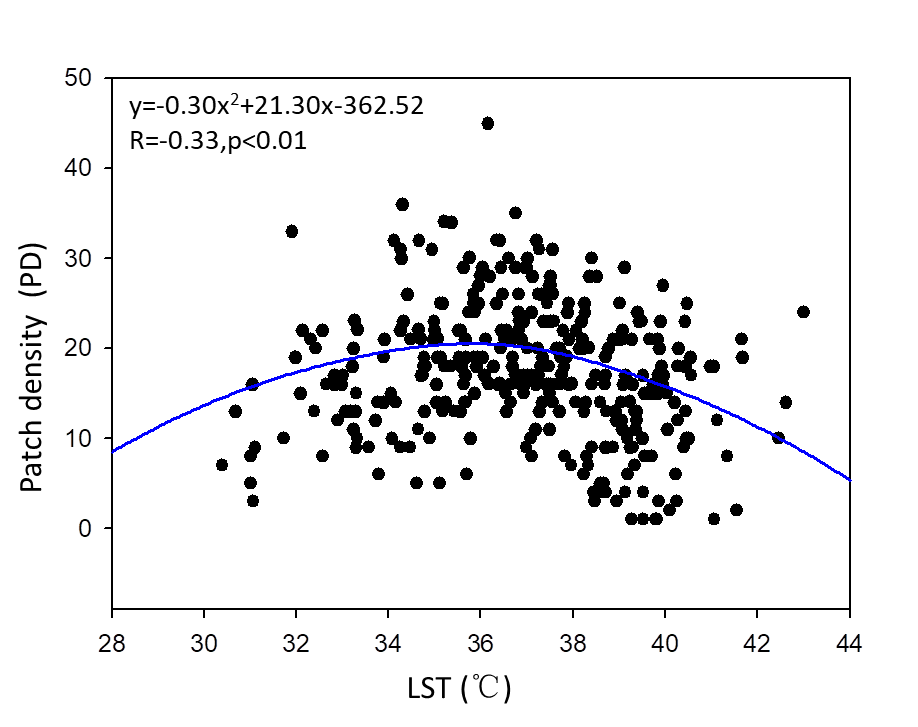

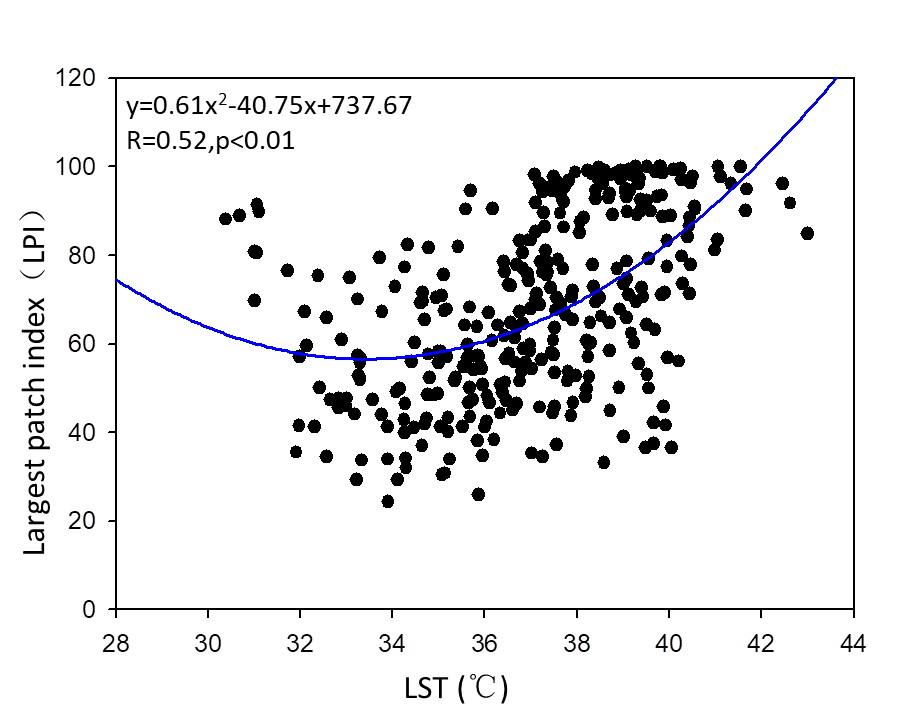


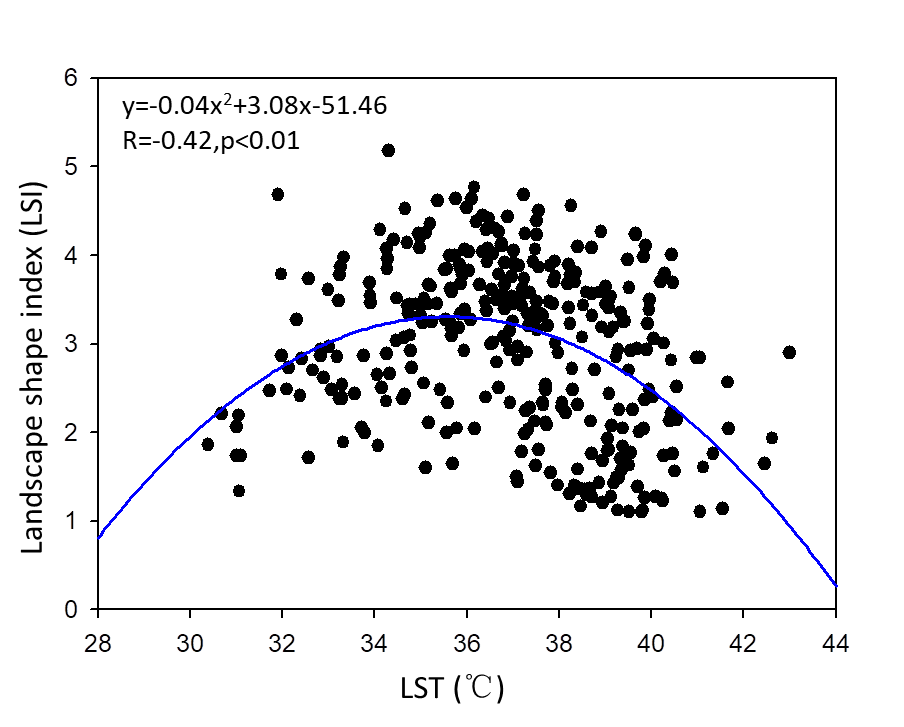

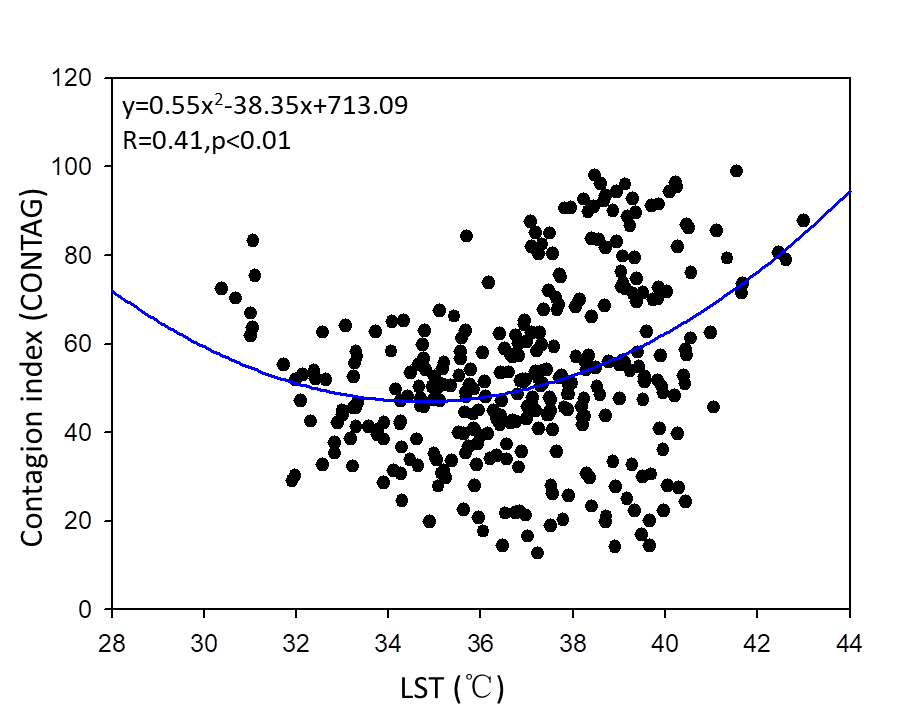


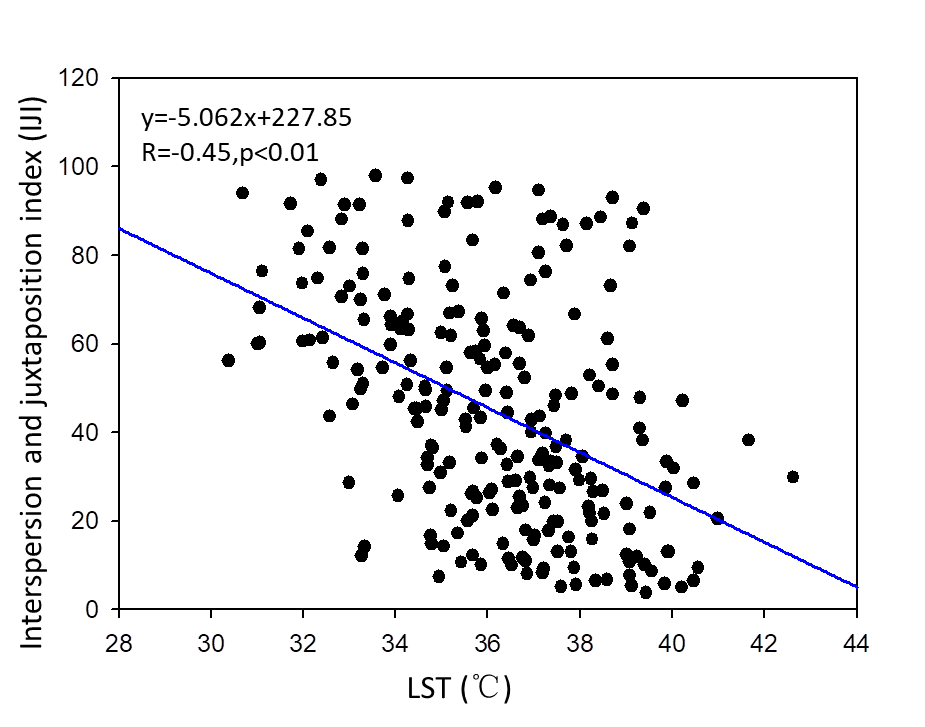

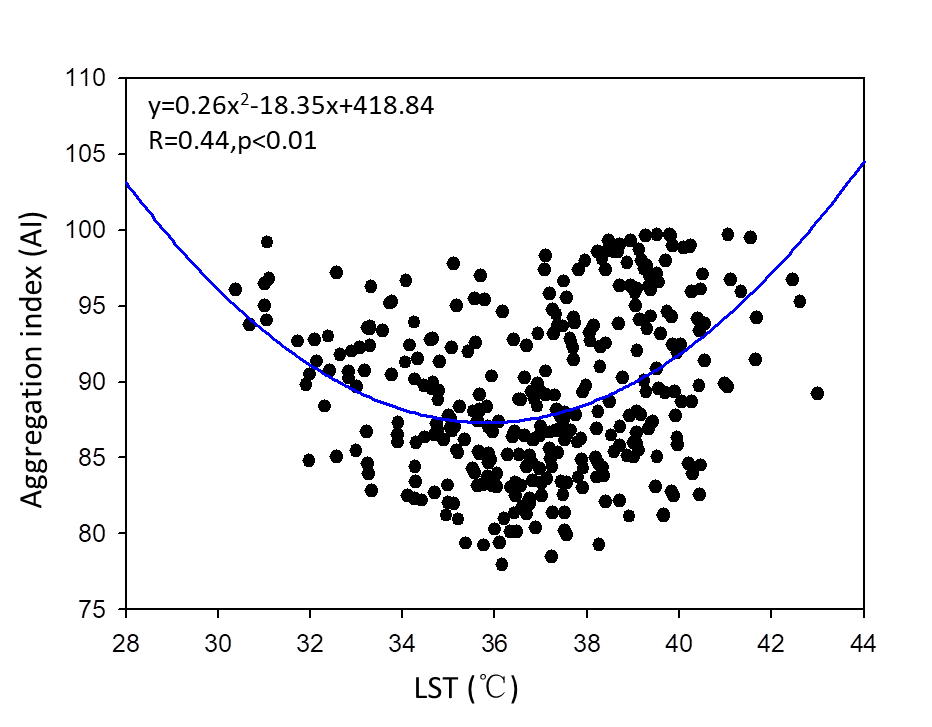


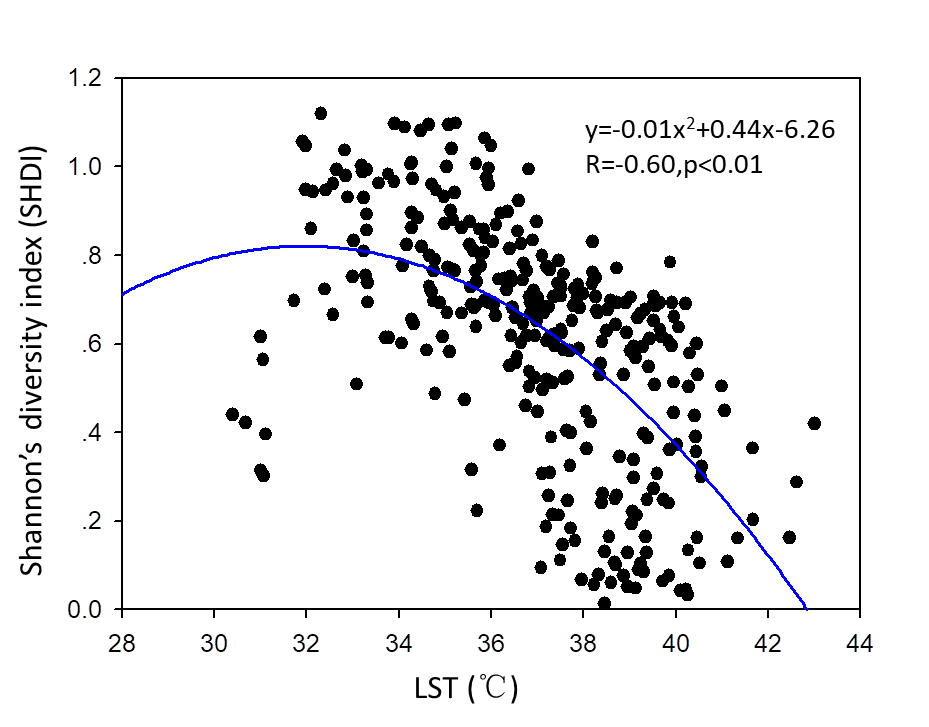

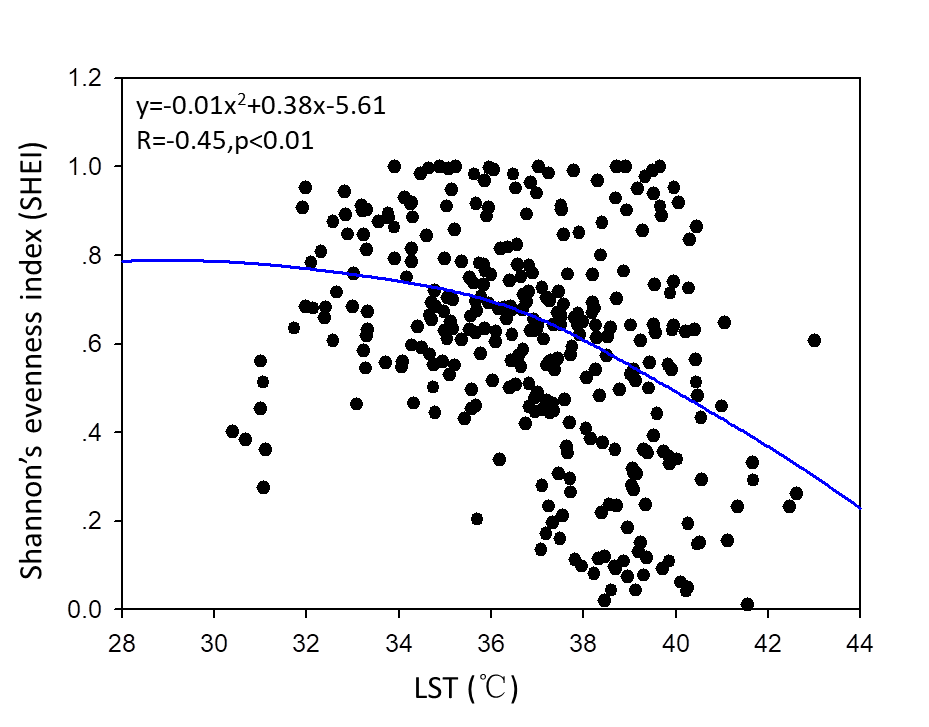


Fig. s5 The correlation between the average LST and landscape level indices
